# Impact of Conventional and Organic Cultivation Methods on Fermentation Efficiency and Volatile Composition of Rye Distillates

**DOI:** 10.3390/molecules31010157

**Published:** 2026-01-01

**Authors:** Maria Balcerek, Katarzyna Pielech-Przybylska, Urszula Dziekońska, Andrea Maria Patelski, Mateusz Różański

**Affiliations:** 1 Institute of Fermentation Technology and Microbiology, Faculty of Biotechnology and Food Sciences, Lodz University of Technology, Wolczanska 171/173, 90-530 Lodz, Poland; katarzyna.pielech-przybylska@p.lodz.pl (K.P.-P.); urszula.dziekonska@p.lodz.pl (U.D.); andrea.patelski@p.lodz.pl (A.M.P.); 2 Polmos Żyrardów Sp. z o.o., Mickiewicza 1-3, 96-300 Żyrardów, Poland; mrozanski@belvederevodka.pl

**Keywords:** rye, organic cultivation, conventional cultivation, fermentation, spirit distillate, volatile compounds

## Abstract

The effect of conditions of rye cultivation (conventional and organic) and method of mash preparation and fermentation, as well as supportive enzyme and yeast strains on the alcoholic fermentation efficiency and chemical composition of the obtained distillates was assessed. The conditions of rye cultivation did not affect the chemical composition of the tested rye grain; however the differences in the fermentation efficiency were observed. The supplementation of mashes from both conventional and organic rye grain with protease had a positive effect on ethanol biosynthesis. The rye distillates contained low concentrations of acetaldehyde (from 22.25 to 34.07 mg/L of 100% *v*/*v* alcohol and met the recommendations for agricultural distillates (<100 mg acetaldehyde/L of 100% *v*/*v* alcohol) in Polish distilleries. The samples obtained from both conventional and organic rye grain, pretreated by the thermal-pressure method, were found to contain higher concentrations of methanol than those obtained by the pressureless method of starch liberation. The concentrations of methanol in all distillates remained below the limit specified in EU Regulation 2019/787 for ethyl alcohol of agricultural origin (i.e., rectified spirit) (≤30 g/hL of 100% *v*/*v* alcohol). The distillates from organic rye grain subjected to pressureless pretreatment contained significantly lower concentrations of 2-methylbutanol and 3-methylbutanol than analogous distillates from conventional rye grain. The digestion of mashes with a protease preparation has been shown to increase the concentrations of 3-methyl-butyl acetate and 2-methyl-butyl acetate in distillates, irrespective of the rye grain type, the processing method, and the yeast strain employed for fermentation.

## 1. Introduction

The spirit industry is one of the most valuable agri-food export sectors in Europe, with distilled spirits encompassing 47 distinct product categories, including a range of geographically specific products that contribute to the cultural identity of their respective regions [1]. In response to the growing consumer demand for food and alcoholic beverages, spirits producers are constantly working to improve the quality of traditional products and to develop new, original spirits. In addition to these efforts, the industry is strongly aware of environmental and social issues, including energy saving, waste management, and the promotion of responsible alcohol consumption. Spirit drink manufacturers are committed to implementing a global strategy of sustainability and responsibility, in line with the United Nations Sustainable Development Goals [2].

Given that spirits production is based on the use of agricultural raw materials, the implementation of sustainable development principles should also take into account the reduction in negative environmental changes caused by agriculture. In the European Union (EU), a set of standards, regulations and incentives contained in the 2019 European Green Deal (EGD) strategy plays an important role in supporting agriculture in its environmental efforts. The EU’s Common Agricultural Policy (CAP) increasingly emphasises the role of institutional activities aimed at meeting society’s needs for the consumption of high-quality agricultural products and the stable and sustainable acquisition of a wide range of environmental goods [3]. One of the most important of these is the action of organic farming. This measure has been a permanent part of the European agricultural policy for many years, aiming to promote an agricultural production system focused on limiting the progressive degradation of the natural environment [4].

According to the Regulation (EU) 2018/848 of the European Parliament and of the Council of 30 May 2018 on organic production and labelling of organic products and repealing Council Regulation (EC) No 834/2007 [5], organic production is defined as an overall system of farm management and food production in line with the preferences of consumers seeking products manufactured using natural ingredients. At the same time, such practices should adhere to the principles of environmental sustainability, promote the preservation of natural resources, and ensure the highest standards of animal welfare. Organic agriculture, as an integrated agricultural production system, relies on processes that ensure the functioning of ecosystems in a sustainable manner, the safety of the food chain, animal welfare, and social justice considerations. Consequently, this generates positive effects on environmental protection, biodiversity conservation, and the production of food that is both sensory and nutritionally superior [6]. However, while conventional agriculture permits the utilisation of chemical fertilisers and pesticides, which can enhance agricultural productivity, organic farming rejects all chemical fertilisers. Consequently, one of the main weaknesses of this production system compared to conventional agriculture is often the lower crop yield. To promote sustainable agriculture, financial support is offered to farmers who voluntarily abandon conventional practices, including chemical pesticides and fertilisers. Organic farming improves food quality and provides numerous environmental benefits [7].

As Macías-Gallardo et al. [8] observed, organically managed farming has the potential to produce grapes of a high-quality suitable for winemaking. It has been demonstrated that grapes harvested from organic vineyards exhibit a superior polyphenol profile in comparison to their conventional counterparts. Specifically, these grapes have been shown to possess elevated concentrations of myricetin, quercetin, resveratrol, chlorogenic acid, and antioxidant capacity in the skin. This can result in enhanced wine quality and sensory attributes [9]. Consequently, the utilisation of organic fertilisers in conjunction with the reduction in pesticide usage in apple orchards has been shown to enhance nutrient content, improve soil health, and reduce environmental impact in comparison with conventional chemical fertiliser treatments. The organic fertiliser treatment also resulted in fruit with significantly higher sugar concentrations and better juice quality, making it a more suitable substrate for cider production [10,11].

The increasing interest in organic products has also been observed in the field of beer production. Barley cultivated for the purpose of organic malting is required to meet the same quality criteria as those used in conventional malting. Acquistucci et al. [12] evaluated eleven barley landraces (*Hordeum vulgare* L.) cultivated under identical organic conditions in order to assess their suitability for organic beer production. The enzymatic activities of α- and β-amylase and the protein modifications during germination (SDS–PAGE) were analysed. Genetic differences among the landraces affected both protein content and amylase activity, thereby influencing malt quality. A protein band at 55–58 kDa showed discriminating ability, thus indicating its potential as a marker for selecting barley suitable for organic beer brewing.

The global spirits market is worth hundreds of billions of USD and is expected to grow further over the next decade, driven by the dynamic growth of the craft segment and increasing consumer interest in organic spirits [13]. Recent economic analyses demonstrate that the organic spirits segment is expanding at a significantly faster rate than the conventional spirits market. Industry reports indicate a sustained double-digit compound annual growth rate (CAGR) for organic vodka, gin, and whisky until 2030. This growth is driven by trends towards premiumisation and a growing demand for the authenticity and traceability of raw materials. This economic momentum underscores the importance of investigating how organic cultivation practices influence the technological parameters and chemical composition of spirit beverages [14].

Despite the increasing commercial importance of organic alcoholic beverages, the scientific research on the direct impact of organic crop production on the quality of distilled spirits remains extremely limited. Current knowledge predominantly stems from studies on wine, cider and beer, in which it has been demonstrated that organic raw materials modify fermentation kinetics, phenolic composition, mineral content and sensory profiles [8,9,10,11,12]. However, no similar investigations have been conducted for cereal-based distillates, leaving a significant research gap concerning the relationship between organic grain cultivation and fermentation efficiency and spirits quality. It is essential to address this gap in order to determine whether the benefits observed in other fermented beverages can be transferred to the spirits sector.

In the spirit beverages industry of EU countries, cereal grains represent the most widely used plant raw material in this sector, including rye grain (*Secale cereale*), which is called the ‘European grain’ [15]. Importantly, rye is well-suited for organic cultivation due to its enhanced winter hardiness, compared to wheat, and its ability to grow on poor, sandy soils where other crops fail. Rye has also been identified as a valuable rotation crop due to its weed suppressive properties, and the cultivation of rye has been shown to enhance the fertility of wasteland and infertile soils. What is more, rye demonstrates superior resilience to pests and diseases when compared with wheat [16]. In relation to the production of organic rye distillates for the manufacture of high-quality spirits, another undisputed advantage of this grain is the relatively high content of phosphorus, potassium, manganese, and calcium, which enables fermentation without the need for supplementation with exogenous mineral nutrients [17]. Research on the quality of winter rye grains cultivated conventionally and organically has shown that organic crops had slightly lower protein and phosphorus content, but higher concentrations of potassium, magnesium, and calcium as compared to traditional crops over a 2-year study period [18].

In EU countries, especially in Poland, rye grain holds significant importance in the production of a wide range of alcoholic beverages, including vodka [19]. However, up until now, in industrial practice, the use of rye grain from conventional cultivation has been predominant. To the best of authors’ knowledge, there has been no research in the literature concerning the effect of organic cultivation of plant raw materials on the quality of spirits.

In the light of the challenges associated with the implementation of organic farming practices and the consumer demand for food and beverages that meet specific nutritional and sensory criteria, the present study aims to address this knowledge gap by conducting a comparative analysis of the fermentation efficiency and chemical composition of distillates derived from rye grain cultivated using conventional and organic methods. Moreover, the study encompassed an assessment of the impact of various factors, including the method of mash preparation and fermentation, the use of a supportive protease preparation, and distinct yeast strains, on the results of alcoholic fermentation and the concentrations of selected congeners in the spirit distillates obtained.

## 2. Results and Discussion

### 2.1. Characteristics of Tested Rye Cultivars and Prepared Sweet Mashes

The chemical composition of rye grain is influenced by cultivar, habitat conditions and agrotechnology [20]. The analysis of the tested raw materials revealed statistically significant differences (*p* < 0.05) exclusively in the moisture content, which amounted to 13.35% *w*/*w* in rye grain from conventional cultivation and 11.60% *w*/*w* in rye grain from organic cultivation. It can be hypothesised that these differences may be due to post-harvest storage conditions rather than cultivation conditions [21]. With regard to protein content (N × 6.25), both tested rye grains exhibited comparable concentrations of this compound from 10.93 to 11.46% d.m. (*p* > 0.05). Furthermore, no statistically significant differences (*p* > 0.05) were observed in reducing sugars and starch contents, which ranged from 5.12 to 5.33 g glucose/100 g and from 48.74 to 50.45 g starch/100 g, respectively (Table 1).

The chemical composition of the sweet mashes was found to vary according to the type of rye grain used as well as the method of its pretreatment and enzymatic hydrolysis (Table 2). Slightly, but statistically significantly (*p* < 0.05), higher extract content, ranging between 16.75 and 17.28 °Blg, was observed in the mashes from the organic rye grain in comparison to those prepared from conventionally cultivated rye (ranging from 16.35 to 16.75 °Blg). Moreover, samples prepared from organic rye grain by the thermal-pressure treatment (according to both SSF and SHF strategies) were characterised by higher extract content than those prepared by the pressureless starch liberation method. In the case of mashes from conventional rye grain, this relation was only observed for the samples prepared by pressure-thermal treatment and hydrolysed according to the SHF strategy.

The concentrations of sugars obtained as a result of the enzymatic hydrolysis of starch were found to vary significantly different among the analysed mashes, with glucose, maltose, and maltotriose being the predominant compounds identified. Initial glucose concentrations were found to be comparatively higher (between 92.3 and 95.6 g/L for mashes from conventional rye grain and between 73.0 and 81.7 g/L for mashes from organic rye grain) in the samples prepared by the pressure-thermal method of starch liberation than in those prepared by the pressureless method (between 43.1 and 58.5 g/L for mashes from conventional rye grain and between 44.9 and 59.2 g/L for mashes from organic rye grain). Furthermore, it can be concluded that the majority of mashes prepared according to the SHF method contain higher amounts of glucose when the method of enzymatic hydrolysis and fermentation (SSF or SHF) is taken into consideration.

In all prepared mashes, the initial concentrations of maltose after the mashing process were found to be significantly lower than those of glucose. Moreover, the concentrations of maltose in the mashes from both types of rye grain were predominantly associated with the method of the raw material pretreatment. Specifically, higher concentrations were observed in samples prepared by the pressureless method (ranging from 17.02 to 26.82 g/L) compared to those obtained by the pressure-thermal method (from 1.42 to 6.05 g/L). Maltotriose was present in the differentiated and relatively low amounts (from 0 to 1.73 g/L). Conversely, the maltotriose concentrations in the mashes prepared by the PLS method exhibited higher levels, irrespective of the type of rye grain (conventional or organic) and the enzymatic hydrolysis methods (SSF or SHF). Statistically significant (*p* < 0.05) differences in dextrins content were identified in all analysed samples (Table 2). However, the method of raw material processing and the strategy of hydrolysis and fermentation had a considerably greater impact than the rye cultivation method. Consequently, the mashes that were prepared according to the PLS and SSF methods contained the highest dextrins contents (on average 82.1 g/L for conventional rye-based mashes and 75.4 g/L for organic rye-based mashes). In turn, samples prepared by the TP method combined with the SSF strategy contained dextrins in amounts ranging from 53 g/L for conventional rye-based mashes to 58 g/L for organic rye-based mashes. Moreover, the implementation of the SHF strategy resulted in a subsequent decrease in dextrin content, particularly in mashes prepared by the PLS method. The observed difference in the quantitative composition of sugars in the tested mashes, depending on the preparation method, is due to the fact that starch gelatinisation occurs during the thermal-pressure treatment, which promotes deeper starch hydrolysis [22]. Conversely, within the SHF strategy, the temperature at which the action of hydrolytic enzymes is optimal is employed for the purpose of starch enzymatic hydrolysis, thereby facilitating the release of fermentable sugars [23].

### 2.2. Characteristics of Fermented Mashes

The analysis of the physico-chemical compositions of all fermented mashes showed the differences in their pH, apparent extract, and sugars concentrations depending on all variables, i.e., rye cultivation conditions, methods of its pretreatment, hydrolysis and fermentation, the addition of the supportive enzyme (protease), and the yeast used for fermentation (Table 3). Moreover, a microbiological analysis of the mashes was conducted, including the determination of the counts of yeast, lactic acid bacteria (LAB), and total mesophilic bacteria (TMB) (Table S3.1).

The pH value of all the sweet mash samples was set to 4.8 [24]. During ethanol fermentation, the pH of the mash drops primarily as a consequence of organic acids production [25]. The lowest pH values, ranging from 3.37 to 3.71, were observed in the mashes fermented by both used yeast strains (SafSpirit HG-1 and Pinnacle Distillers Yeast), from rye grain of organic cultivation, prepared by the PLS method both in combination with the SSF and SHF strategies. The aforementioned mashes exhibited relatively higher (*p* < 0.05) values of apparent extract (determined in the presence of alcohol) in addition to unfermented sugars, predominantly glucose and maltose. Concurrently, statistically significantly (*p* < 0.05) higher levels of glycerol, and organic acids, including lactic acid, and acetic acid, were observed in these samples (Table 3).

It should be noted that the critical point of pressureless starch-liberation methods is the risk of microbiological contamination [24]. Microbial analysis of mashes prepared from organically cultivated rye grain using the PLS method, before fermentation, showed counts of lactic acid bacteria (LAB) and total mesophilic bacteria (TMB) of 1.50 ± 0.20 log cfu/mL and 1.65 ± 0.15 log cfu/mL, respectively. In turn, analogous mashes prepared using the TP method contained levels of LAB and TMB below 1.00 log cfu/mL (Table S3.1).

The LAB and TMB counts in the organic rye grain-based mashes prepared using the PLS method increased significantly (*p* < 0.05) after fermentation. The respective ranges were 3.50 ± 0.50 to 4.60 ± 0.20 log cfu/mL and 3.65 ± 0.40 to 4.65 ± 0.23 log cfu/mL. Moreover, a decrease in yeast growth was observed in these samples. The use of the TP method for mash preparation resulted in significantly lower (*p* < 0.05) LAB and TMB contamination after fermentation In turn, the LAB and TMB counts in fermented mashes prepared from conventionally cultivated rye using the PLS method were significantly lower, not exceeding 1.18 ± 0.06 and 1.60 ± 0.25 log cfu/mL, respectively (Table S3.1). It can be assumed that organic grain has a more diverse microflora than conventionally grown grain, since no pesticides or seed treatments are used. It may harbour LAB strains that are more resistant to fermentation stresses. A study by Lori et al. [26] showed differences in microbial counts and activities between organic and conventional farming systems, demonstrating that organic farming enhances total microbial abundance and activity in agricultural systems.

According to Broda and Leja [27], the initial contamination of distillery mashes depends on the type and quality of the raw material and the processing conditions. It may range from 2 to 6 log cfu/mL for LAB and 3–6 log cfu/mL for TMB. After fermentation, the number of microorganisms can increase, particularly under industrial conditions, reaching 5–8 log cfu/mL (LAB) and 6.5–8 log cfu/mL (TMB).

Regarding the fermentation by-products determined in the tested rye mashes, glycerol is a compound that plays a crucial role in protecting yeast against environmental stressors [28]. In turn, lactic acid and acetic acid may act synergistically to reduce ethanol production by yeast in distillery mashes [29]. The results obtained in our study are consistent with the findings of Pielech-Przybylska et al. [24], who observed that when the pH of fermented distillery mashes decreased from 4.8 to 3.3–3.7, there was an increase in lactic acid concentration and lactic acid bacteria growth.

The remaining fermented rye mashes, from both conventional and organic grains, were characterised by a higher pH, ranging from approximately 4.0 to 4.39, which is in line with the results of our previous study on the fermentation of mashes prepared from rye and barley grains, and the corresponding malts that served as sources of amylolytic enzymes and starch [19]. No clear influence of the methods of mash preparation and fermentation, as well as the yeast strains on the pH values of mashes after fermentation completion was observed. Furthermore, the tested mashes exhibited varying quantities of unfermented sugars (glucose, maltose and maltotriose) as well as dextrins. However, despite these differences in sugars content, the intake of total sugars, calculated from the difference in the content of total sugars in mashes before and after the fermentation, was high in all mashes and ranged from approximately 94% to 99%.

When assessing fermentation efficiency (Table 4), it was observed that the processing of grain from conventional cultivation by the PLS method associated with both the SSF and SHF strategies resulted in significantly higher (*p* < 0.05) ethanol concentrations (from 8.66 to 9.25% *v*/*v*) and process efficiency (from 87.31 to 92.57% of the theoretical) in most samples in relation to the analogous samples from organic rye grain (ethanol content from 7.52 to 8.70% *v*/*v*, fermentation efficiency from 75.05 to 87.04% of the theoretical). With regard to the mashes prepared by the thermal-pressure method, the aforementioned relationship has not been fully confirmed. However, irrespective of the type of rye and the methods of its pretreatment, hydrolysis and fermentation, it was observed that the supplementation of mashes with protease had a positive effect on ethanol biosynthesis and fermentation efficiency. In turn, regarding the fermentation activity of the yeast, it is noteworthy that despite the observed differences in ethanol concentrations within the mashes, both the applied strains (SafSpirit HG-1 and Pinnacle Distillers Yeast (S)) facilitated an efficient fermentation process and obtained indicators that are comparable to those obtained during the processing of grain raw materials commonly used in the distilling industry for the production of ethanol [19,30].

### 2.3. Chemical Composition of the Distillates Obtained

During the fermentation process, yeast produces volatile compounds in addition to ethanol and carbon dioxide. These include organic acids, carbonyl compounds, alcohols and esters, which have a differentiated impact on the flavour and aroma of spirits [31].

The obtained rye spirit distillates were characterised by an acidity level ranging from 1062 to 1564 mg acetic acid/L alcohol 100% *v/v* (Table 5). Both the minimum and maximum acetic acid concentrations in this range concern the distillates obtained from conventionally cultivated rye grain. The highest acidity was found in the distillate obtained after fermentation of mashes prepared using the PLS-SHF method, while the lowest was observed in those prepared using the TP-SSF method, both treated with amylolytic enzymes and fermented with the SafSpirit HG-1 yeast strain. This relationship was not observed in the samples of distillates from organic rye grain. The acidity of the organic rye distillates ranged from 114.30 to 135.42 mg/L of 100% *v*/*v* alcohol. There were no statistically significant differences between the majority of the samples.

The methods used for the pretreatment of raw materials, hydrolysis and fermentation can have a significant impact on the chemical composition of the resulting distillates, including their acidity [19]. The temperature of 90 °C used in a pressureless starch liberation method inactivates solely vegetative forms of microorganisms. Moreover, separate hydrolysis in the SHF method carried out at a temperature of 65 °C has been shown to favour activation of heat-resistant spores [24], which may affect the efficiency of the process and the quality of the spirit. Regulation (EU) No. 2019/787 [1] does not specify the limit for acidity in the distillates of agricultural origin. In accordance with the stipulations set out in the Polish regulation [32], the acidity of agricultural distillates, expressed in grams of acetic acid per litre of alcohol 100% *v*/*v*, should not exceed 80 mg/L for spirits derived from rye and potato. Depending on the intended use of the obtained rye distillates, there may be a need to eliminate volatile congeners.

The compounds which may have a negative effect on the quality of spirits are carbonyl compounds, i.e., aldehydes and ketones. The concentrations of carbonyl compounds in agricultural distillates depend on the quality of raw materials, their chemical composition, the conditions of technological processes, and microbial contamination [33]. The main compound within this group, i.e., acetic aldehyde, ranged from approximately 22 to 33 mg/L of 100% *v*/*v* alcohol in the rye distillates tested, both from conventional and organic cultivation. This concentration met the requirements for acetic aldehyde in agricultural distillates (<100 mg/L of 100% *v*/*v* alcohol) outlined in the Polish regulation [32]. In addition, despite the presence of statistically significant differences in acetaldehyde content among the samples, no direct impact of the methods of starch liberation and hydrolysis, along with the supportive enzyme (protease) and yeast strains used was observed. Moreover, the rye distillates obtained in this study were found to contain varying (*p* < 0.05) concentrations of acetone, and 2,3-butanedione, 3-methylbutanal, 2-methylbutanal, and phenylacetaldehyde which are Maillard reaction and Strecker degradation compounds [34]. Notably, 2,3-butanedione (diacetyl) concentrations were found to be particularly high, at levels of several tens of mg/L, in certain distillates from both organic and conventional rye grain that had been pretreated by thermal-pressure cooking and then digested by amylolytic enzymes and protease.

Diacetyl, a butter-tasting vicinal diketone, is primarily produced as a by-product of amino acid metabolism, notably during the biosynthesis of valine and isoleucine biosynthesis, by yeast and lactic acid bacteria during alcoholic and malolactic fermentation. It is formed during the conversion of pyruvate to α-acetolactate, which is then non-enzymatically oxidised to diacetyl outside the cell [35,36]. One possible reason for α-acetolactate secretion is to protect the yeast from carbonyl stress [37]. The general FAN content of the wort may also affect the valine uptake rate, which consequently affects diacetyl production. Krogerus and Gibson [36] reported that when FAN levels were lowered, the diacetyl production was also lowered, presumably due to the faster absorption of preferred amino acids resulting in an earlier and greater demand for valine, which is then taken up more readily due to less competition for permease interactions. Increasing the initial wort’s background levels of amino acids (while keeping the valine concentration constant) resulted in greater diacetyl production. Moreover, the oxidation of unsaturated fatty acids during the thermal treatment may lead to the generation of α-dicarbonyl compounds such as glyoxal, methylglyoxal, and 2,3-butanedione [38].

Methanol is a compound that is present in spirits derived from plant raw materials. This compound is not a by-product of yeast activity during fermentation, rather it is mainly liberated from pectic substances by enzymatic degradation under the influence of a pectin methylesterase, particularly during the fermentation process [39]. In the present study, the rye distillates obtained from both conventional and organic rye grains pretreated by the thermal-pressure method were found to contain higher concentrations of methanol than those obtained by the pressureless method of starch liberation. This observation was particularly pronounced in the distillates derived from organic rye grain, where the methanol concentration was the highest and ranged from 82.63 to 112.64 mg/L of 100% *v*/*v* alcohol (Table 6). The results of our study are in line with those reported by Pielech-Przybylska et al. [24] who also observed a higher methanol concentration in spirits obtained from starchy raw materials-based mashes prepared by the thermal-pressure method (approx. 150 °C) in comparison to the pressureless method (90 °C). No evident relationship was observed between the addition of the supportive enzyme (protease) and the yeast strains in terms of their effect on methanol content. However, it should be noted that all the rye distillates obtained complied with the EU regulation stipulating that the maximum permitted methanol content in ethyl alcohol of agricultural origin (i.e., rectified spirit), even though they were not purified. This equates to 30 g/hL of 100% *v*/*v* alcohol (equivalent to 300 mg/L) [1].

From a quantitative point of view, a significant group of fermentation by-products are higher alcohols, which are primarily represented by 1-propanol, 1-butanol, 2-methyl-1-butanol and 3-methyl-1-butanol. These compounds have been demonstrated to play an important role in the formation of flavour qualities in spirits such as whisky and others [31]. The content of higher alcohols is strongly associated with the kind of raw material, the concentrations of polysaccharides in fermentation media, the assimilable nitrogen availability, and different rates of their assimilation by a particular distiller’s yeast [40]. It was observed that distillates from conventional rye grain subjected to pressureless pretreatment contained significantly higher (*p* < 0.05) concentrations of 2-methylbutanol and 3-methylbutanol (ranging from 1415.50 to 3300.92mg/L of 100% *v*/*v* alcohol) compared to analogous distillates from organic rye grain (ranging from 612.26 to 701.20 mg/L of 100% *v*/*v* alcohol). This relationship was not confirmed for the distillates from mashes after pressure-thermal treatment. The observed differences may be related to variations in the composition or activity of amino acid precursors in conventional and organic rye grains, or to the enzymes involved in the formation of higher alcohols. The results of our previous study confirm that the type of raw material and preparation method influence the content of higher alcohols. Vashishtha et al. [41] found that terroir, i.e., specifically seasons (year) and geography (location), may also affect the chemical characteristics of wheat-based Irish whiskey. Moreover, literature data [42,43,44] indicate that the elemental composition of rye grains used to prepare distillery mashes influences yeast metabolism and biochemical processes during fermentation, which can affect fermentation performance and, consequently, the profile of volatile compounds.

Interesting observations were made when assessing the effect of protease treatment of mashes on the concentrations of higher alcohols. The majority of spirits obtained from rye mashes digested by this supportive enzyme contained lower concentrations of isoamyl alcohols than spirits obtained from mashes treated only by amylolytic enzyme preparations. This phenomenon can be attributed to the observation that an elevated initial concentration of assimilable nitrogen, resulting from protease activity, leads to a reduction in the production of higher alcohols by yeast. This is due to the rapid conversion of most keto acids to their respective amino acids [45]. In turn, 2-propanol concentrations exhibited an inverse correlation, and the application of protease to mashes resulted in the stimulation of its biosynthesis, which was observed in all tested distillates (Table 6). However, no close relationship was observed between 2-propanol content and rye cultivation, starch liberation methods, hydrolysis and fermentation systems and the yeast strains used for fermentation. According to the literature [46,47], 2-propanol is synthesised via the reduction in acetone catalysed by alcohol dehydrogenase. For acetone synthesis, in turn, two molecules of acetyl-CoA condense to form one molecule of acetoacetyl-CoA (by acetoacetyl-CoA synthase). This then forms acetoacetate (by acetoacetyl-CoA transferase) which is finally decarboxylated to form acetone (by acetoacetate decarboxylase) [46]. The synthesis of 2-propanol during the alcoholic fermentation of distillery sweet mashes can be affected by differences in the methods used for sweet mash production, as well as the complexity of the biochemical processes occurring during fermentations involving yeast and other microorganisms (mainly lactic acid bacteria) [48].

Esters produced during the fermentation and distillation processes also significantly influence the organoleptic features of spirits. The presence of these compounds has been attributed to the activity of yeast esterases during the fermentation process [49]. The analysis of the qualitative and quantitative composition of esters in the studied rye distillates showed that ethyl acetate predominated (from 109.15 to 223.05 mg/L of 100% *v*/*v* alcohol), which is in agreement with our previous report [19] on the chemical composition of distillates based on starchy raw materials. No strict relationship was observed between the rye cultivation conditions and the methods of starch liberation and hydrolysis, as well as the addition of the supportive enzyme (protease), the yeast strains and the ethyl acetate content (Table 7). Other acetate esters present were: isobutyl acetate, 2-methylbutyl acetate, and the dominant 3-methylbutyl acetate, also known as isoamyl acetate. Its highest concentrations (between 3.71 and 5.63 mg/L of 100% *v*/*v* alcohol) were found in the distillates obtained from conventional rye grain processed by the PLS method of starch liberation, both associated with the SSF and SHF strategies. Moreover, protease treatment was found to significantly increase the concentration of this compound (*p* < 0.05) in all tested distillates, regardless of the type of rye grain, processing method or yeast strain used. A similar trend was observed for the concentrations of ethyl octanoate, ethyl decanoate, and ethyl tetradecanoate in all the samples from organic rye and in some samples from conventional rye, particularly those from mashes prepared by the PLS method. It can be assumed that although the primary role of proteases is the breakdown of proteins into peptides and amino acids, they may also be involved in the biosynthesis of esters [50]. The most influential factors in producing higher ethyl esters during the alcoholic fermentation of plant materials are the availability of fatty acid precursors, the selection or genetic modification of yeast strains, the fermentation temperature and the balance of unsaturated fatty acids [51,52]. Rye grain is rich in unsaturated fatty acids such as linoleic acid (18.9–54.0%), oleic acid (20.6–37.9%), palmitic acid (10.8–22.4%), and linolenic acid (2.4–8.3%) [53,54]. Although organic and conventional cultivation may cause minor differences, genotype and environment generally have a greater influence on fatty acid composition than the farming system itself [55].

From an applied perspective, the results of our study suggest that combining organic raw materials, targeted enzyme supplementation and controlled mash processing can be used to tailor the volatile profile and sensory characteristics of rye distillates. Importantly, optimising nutrient availability and mash processing can simultaneously improve fermentation efficiency and ensure compliance with regulatory limits for volatiles. This links the use of sustainable raw materials with the optimisation of industrial processes and EU sustainability objectives [56]. However, as the study was conducted using a single variety of rye, further research involving genetically diverse rye cultivars is needed to confirm the applicability of these findings more broadly.

### 2.4. The PCA and Hierarchical Cluster Analysis

Principal component analysis (PCA) was used to describe the variability in the volatile compound profiles of distillates obtained from rye grown under two cultivation systems (conventional and organic). The distillates were produced using different mash preparation methods (TP and PLS), hydrolysis and fermentation approaches (SSF and SHF), with and without protease addition, and two commercial distillers’ yeast strains. The influence of these factors was assessed indirectly, based on the distribution of the cases (distillate samples) in the principal component space. In the initial phase of the analysis, the principal components (PC) were extracted, which significantly contributed to explaining the total variance of all the parameters studied. For this purpose, a double criterion was employed, incorporating a scree plot and eigenvalues > 1. The PCA demonstrated that the first four principal components collectively account for approximately 72% of the total variance of the data (Supplementary Data, Table S8). To provide a more comprehensive interpretation that goes beyond the scope of descriptive PCA, the relationships revealed by the loadings were evaluated in the context of known biochemical and technological pathways influencing the formation of volatile compounds in cereal-based fermentations [24,51,57]. This approach enables the principal components to be linked directly to metabolic and process-dependent mechanisms, rather than to statistical variation alone.

**Figure 1:**
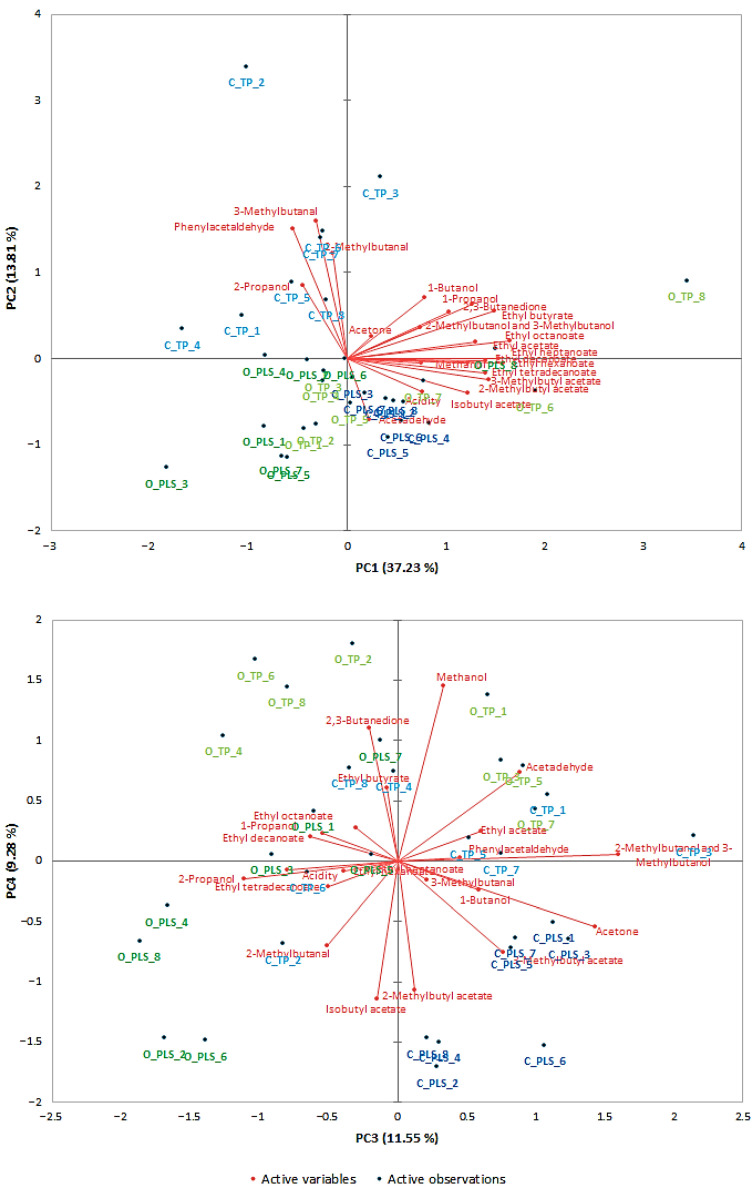
Principal component analysis (PCA) biplot for tested distillates. Red vectors represent volatile compounds, while blue and green points correspond to distillates produced from conventional (C) and organic (O) rye grain, respectively.

The projections of the variables and the cases onto the plane of factors PC1 and PC2, and PC3 and PC4 are presented in Figure 1. Factor 1, corresponding to the largest eigenvalue of 8.19 (Table S8), demonstrated a strong positive correlation with esters such as ethyl acetate, ethyl butyrate, isobutyl acetate, 3-methylbutyl acetate, 2-methylbutyl acetate, ethyl hexanoate, ethyl heptanoate, ethyl octanoate, ethyl decanoate and ethyl tetradecanoate, as well as with 1-propanol, while a moderately positive correlation was observed with 2,3-butanedione and acidity. These esters are grouped in PC1, which is consistent with their shared biosynthetic origin. Medium- and long-chain ethyl esters are form through from the esterification of ethanol with fatty acyl–CoA intermediates, a reaction catalysed by yeast alcohol acyltransferases (AATs). Production of these esters increases when yeast cells exhibit enhanced fatty-acid turnover, or when technological steps such as thermal-pressure treatment increase lipid availability in the mash [24,51]. Therefore, PC1 can be interpreted as mechanistic axis reflecting the efficiency of ester formation pathways during fermentation. Factor 2 was found to be associated with five volatile compounds. A strong positive correlation was observed for 2-methylbutanal, 3-methylbutanal and phenylacetaldehyde, while a moderate correlation was observed for 2-propanol. In turn, a moderate negative correlation was observed for acetaldehyde. These aldehydes are characteristic intermediates of the Ehrlich pathway, formed during the catabolism of branched-chain and aromatic amino acids [57]. Their loadings on PC2 therefore suggest that this component reflects variations in amino acid availability and yeast metabolic activity. This variation could be due to the addition of protease, differences in mash pretreatment (TP vs. PLS) or the composition of raw materials, all of which influence the release of amino acid precursors. The distribution of cases on the PC1 vs. PC2 graph (Figure 1) indicates that the profile of volatile compounds depends on the combination of technological factors used in the study. Observations representing distillates obtained from organic rye grain using the thermal-pressure method (O_TP_8, O_TP_7, and O_TP_6) and the pressureless starch liberation method (O_PLS_8) are distributed in the area of high positive PC1 values, which reflects the high content of higher fatty acid ethyl esters in these samples. This is in line with the literature evidence that thermal processing can disrupt cell structures and increase the release of lipid precursors, thereby promoting ester synthesis during fermentation [24]. In contrast, samples of distillates obtained from organic rye grain (PLS-SSF method, without protease, Pinnacle Distillers yeast) and from conventional cultivation (TP-SSF method, treated with protease, Pinnacle Distillers yeast), marked as O_PLS_3 and C_TP_4, respectively, correspond to cases with extremely negative values on PC1, which can be explained by the fact that most of these compounds were not detected in these samples.

With regard to PC2, positive values were established for observations representing distillates obtained from conventionally cultivated rye, pretreated by the TP method. The samples designated as C_TP_2 and C_TP_3 (SSF) achieved the highest values, which suggests a high concentration of variables positively correlated with this component, i.e., 2-methylbutanal, 3-methylbutanal and phenylacetaldehyde [58,59]. This pattern confirms that aldehydes can be produced through pressure-thermal treatment, in which they form from amino acids via Strecker degradation, or through the fermentation process, in which they form as a result of amino acid catabolism in the Ehrlich pathway [57,60,61]. Positive values in PC2 are also demonstrated by observations representing samples C_TP_5, C_TP_6 and C_TP_7, in which elevated concentrations of the aforementioned compounds were also determined. In the case of organic rye grain, the use of the same method did not lead to such a clear shift towards high PC2 values, suggesting that the effect of the TP method on the quantitative profile of aldehydes depends on the type of raw material. Observations corresponding to samples designated as O_PLS_5 and O_TP_1 (organic rye), which exhibited negative values on the PC2 axis, were characterised by the lowest concentrations of these compounds.

The third and fourth factors (Table S8) explain 11.6% and 9.3% of the total variance, respectively. Factor 3 demonstrated a strong positive correlation with 3-methylbutanol and acetone, while a moderate correlation was observed with acetaldehyde. These findings suggest that samples with high values in PC3 (C_TP_3, C_PLS_3 and C_PLS_1) contain high concentrations of these compounds. As fusel alcohols such as 3-methylbutanol are by-products of the Ehrlich pathway, it is likely that PC3 captures secondary metabolic shifts related to amino acid catabolism and the carbon–nitrogen balance during fermentation [57]. In turn, Factor 4 demonstrated a strong positive correlation with methanol and a moderate correlation with 2,3-butanedione, while exhibiting a moderate negative correlation with isobutyl acetate and 2-methylbutyl acetate. With regard to PC4, elevated values were characterised by observations representing samples obtained from organic rye subjected to TP treatment prior to the enzymatic hydrolysis process (O_TP_6 and O_TP_2). This supports the interpretation that thermal-pressure processing may intensify the release of methanol by accelerating the breakdown of pectin or other structural polysaccharides [62].

**Figure 2:**
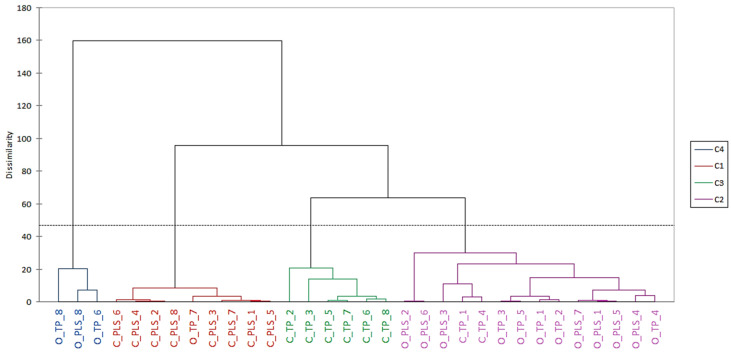
Dendrogram from hierarchical clustering analysis (HCA) of tested rye distillates. The dotted horizontal line indicates the selected dissimilarity (distance) threshold used to cut the dendrogram and define the main clusters.

The hierarchical cluster analysis (AHC) was used to highlight the interaction between the technological factors studied. The AHC results are presented in the form of a dendrogram (Figure 2), which shows four main clusters. The presence of four distinct clusters suggests that both agricultural practices and mashing and fermentation conditions exert measurable and systematic effects on aroma compound formation. Cluster 1 (C1) comprises nine distillate samples, eight of which were obtained from conventionally cultivated rye. Levels of acetone and esters of acetic acid and higher alcohols were found to be higher in comparison with the other clusters. Conversely, cluster 3 (C3) comprised distillates obtained from conventionally cultivated rye (six samples), prepared using the TP method, which were distinguished by elevated concentrations of aldehydes: 2-methylbutanal, 3-methylbutanal, and phenylacetaldehyde. This is in line with results of the study by Pielech-Przybylska et al. [63], which confirmed that the thermal-pressure treatment of rye grains before fermentation increased the content of Strecker aldehydes in the resulting mash.

Cluster 4 (C4) grouped only three samples (O_TP_6, O_TP_8, and O_PLS_8), i.e., distillates from organically grown rye (samples with the highest positive PC1 values). This indicates that a high level of higher fatty acid ethyl esters (which was even four times higher than in the remaining distillates) is characteristic of a specific combination of organic rye and the method of mash preparation and fermentation. The specificity of this cluster indicates a unique chemical ‘fingerprint’ associated with organic raw material combined with particular processing conditions. This strong clustering effect highlights the interplay between organic cultivation and fermentation strategy. It suggests that organic rye may contain different lipid or fatty acid precursors that promote esterification. While literature data suggest that organic cultivation can influence the lipid and fatty acid composition of cereal grains, the effect is inconsistent across studies and cereal types. Some studies show that organic fertilisers can alter the levels of key fatty acids (such as palmitic, oleic, linoleic, and linolenic acids), which are important precursors for esterification reactions in plant metabolism [64,65]. However, other studies indicate that environmental factors and cereal genotype have a greater impact on fatty acid profiles than the cultivation system itself, with no uniform differences observed between organic and conventional methods [66].

Finally, cluster 2 (C2), encompassing more than 40% of all samples, was characterised by comparatively low levels of esters such as 2-methylbutyl acetate, 3-methylbutyl acetate, and phenylacetaldehyde. The broad inclusion of distillates in this cluster suggests that many production variants lead to more moderate volatile profiles, and this group may represent a technologically ‘neutral’ aromatic category.

## 3. Materials and Methods

### 3.1. Raw Materials

Rye grain of the cultivar Dańkowskie Złote, obtained from conventional and organic cultivation, was used as the starchy raw material. The crops were harvested in 2023 from fields located at the Bartężek Agricultural Farm Sp. z o. o. (Warmia-Masuria Voivodeship, Poland, 53.82149° N, 19.86911° E). The conventional cultivation regime involved the application of mineral fertilisers, i.e., ammonium nitrate, ammonium phosphate, and potassium chloride. Moreover, the herbicide treatment was applied. In contrast, the cultivation of organic rye was carried out in accordance with the principles of organic production, thus avoiding the use of mineral fertilisers and herbicides (certificate no. PL-EKO-01.616-0010289.2024.001). The harvesting of conventional and organic rye grain was carried out in parallel, using separate machinery and equipment, to avoid cross-contamination. After harvesting, the rye grain from traditional and organic farming was stored in separate silos under identical, controlled conditions. The storage temperature was offset to 13–15 °C and the grain’s moisture content at the time of storage did not exceed 14%, which is consistent with the recommended safe limits for rye grain [67]. Prior to sampling for the study, rye grain was probed at multiple depths and locations within each silo. The collected increments were thoroughly combined to obtain representative composite samples, minimising the risk of batch-to-batch variability.

### 3.2. Enzymatic Preparations

The following amylolytic and supportive enzyme preparations (Novozymes A/S, Bagsværd, Denmark) were used during the preparation of the mashes:

LpHera^®^ (α-amylase, EC 3.2.1.1), at a dose of 0.50 mL/kg starch,

Saczyme^®^ Plus (glucan 1,4-α-glucosidase, EC 3.2.1.3), at a dose of 0.72 mL per 1 kg of starch,

Viscoferm^®^ (a multienzyme complex containing: cellulase, EC 3.2.1.4; xylanase (endo-1,4-), EC 3.2.1.8; and β-glucanase (endo-1,3(4)-), EC 3.2.1.6), at a dose of 0.15 mL/kg raw material,

Neutrase^®^ (neutral protease, EC.3.4.24.28), at a dose of 0.1 mL/kg raw material.

### 3.3. Yeast

Fermentation was carried out using the dry distillery yeast strains (*S. cerevisiae*): —SaftSpirit^TM^ HG-1 dry distillery yeast (Fermentis by Lesaffre, Marcq-en-Barœul, France), which has been designed to produce a high ethanol concentration with a broad range of applications across various process conditions (pH range—not specified, temperature range 25–35 °C) and with diverse substrates [68].

Pinnacle Distillers Yeast (S) (AB Biotek, London, UK), which has been designed to produce high ethanol titres with a broad range of applications, under various process conditions (pH range 4.0–5.0, temperature range 33–37 °C) and using various substrates [69].

### 3.4. The Course of Experiments

Two methods were employed for the preparation of sweet mashes: pressureless liberation of starch (PLS) and thermal-pressure (TP) method. Both processes were performed in two variants, i.e., with separate hydrolysis and fermentation (SHF) and with simultaneous saccharification and fermentation (SSF) [70].

In order to prepare sweet mashes according to the PLS method, the milled rye grain was mixed with tap water (3.5 L of water per 1 kg of milled rye grain) in a vessel placed in a water bath and equipped with a laboratory stirrer and thermometer. The mixture was subjected to continuous stirring and heating until it reached a temperature of 50 ± 1 °C. Subsequently, the viscosity-reducing Viscoferm^®^ preparation was added. The mixture was stirred continuously and heated to 90 ± 1 °C, and digested with LpHera^®^ preparation for 60 min to achieve liquefaction of the mixture. Following this, the mixture was divided into two equal portions. One portion of the mash was prepared according to the SHF (Separate Hydrolysis and Fermentation) strategy. This portion was cooled to a temperature of 65 ± 1 °C and subjected to saccharification with the Saczyme^®^ Plus preparation, either alone or with a supportive Neutrase^®^ preparation. The mixture was then kept for 30 min at this temperature and then cooled to a temperature of 30 °C, i.e., optimal for yeast inoculation.

The second portion of the mash, which was prepared according to the SSF (Simultaneous Saccharification and Fermentation) strategy, was cooled to 65 ± 1 °C and digested with the same doses (as in the case of the SHF variant) of saccharifying Saczyme^®^ Plus preparation, without or along with a supportive Neutrase^®^ preparation. The temperature was then immediately reduced to a level suitable for yeast inoculation, i.e., 30 °C. Prior to the introduction of the yeast inoculum, all prepared mashes were acidified with a sulfuric acid solution (25% *w*/*v*) from pH 5.6–5.8 to 4.8 [24]. In trials not involving protease treatment, an aqueous solution of (NH_4_)_2_HPO_4_ (0.2 g/L mash) was supplemented to provide the necessary nutrients for yeast growth.

In the case when the thermal-pressure (TP) method being employed, 5 kg of rye grain was placed in a tapered cylindrical steamer that had been previously filled with 17.5 L of water heated to boiling point. The steamer was then closed and the rye grain was steamed at 150 °C with a pressure of 0.4 MPa for 35 min, with periodic circulation of the content. After completion of the pretreatment of the rye grain, the content of the steamer was subsequently transferred to a cylindrical steel mashing vessel equipped with a heating/cooling coil and a thermometer. When the mash had been cooled to the temperature of 90 ± 1 °C, it was digested with enzyme preparations used in the PLS method according to the SHF and SSF strategies. Next, an adjustment of pH, and a supplementation with nutrients were carried out in the same way as in the PLS method.

Prior to the inoculation of the rye mashes, yeast at a dose of 0.5 g per 1 L of mash was hydrated and disinfected (15 min incubation of cells suspended in 25% *w*/*w* sulfuric acid solution, pH 2.5, at room temperature). The yeast cream was then added to the mash (without neutralisation). The inoculated mashes were mixed carefully. The fermentation process was then conducted for a period of three days at a temperature of 32 °C in 2 L glass bottles containing 1.2 L of mash. The bottles were closed with fermentation locks containing paraffin oil. The course of the process was controlled gravimetrically.

After completion of fermentation, the ethanol was distilled from the mashes using a laboratory distillation unit. The raw distillates were then strengthened to approximately 43% *v*/*v* in a distillation apparatus equipped with a dephlegmator according to Golodetz, prior to analysis [71].

### 3.5. Analytical Methods

The rye grain analysis entailed the determination of moisture (using a Radwag WPS-30S Moisture Analyzer, Radwag, Radom, Poland); protein content (N × 6.25), using the Kjeldahl method; and sugar content, using the HPLC technique as described by Dziekońska-Kubczak et al. [72].

The analysis of mashes (sweet and fermented) included the determination of extract content (total in sweet mashes and apparent in fermented mashes), expressed in Balling degrees (°Blg), using an oscillatory densitometre DMA^TM^ 1001 (Anton Paar GMBH, Graz, Austria), pH (pH-meter HandyLab, SI Analytics, Mainz, Germany) and content of sugars, ethanol as well as other fermentation by-products through HPLC [72].

The microbial analysis of mashes included the determination of yeast [73], lactic acid bacteria (LAB), and total mesophilic bacteria (TMB) [74]. Samples of mash were prepared for microbial analysis according to ISO 6887 [75]. The limit of detection for the above enumeration techniques was 10 cfu/mL. The results were expressed as log cfu/mL.

The acidity of spirit distillates was determined by the titrimetric method [76] with a use of 0.1 M sodium hydroxide solution and phenolphthalein as an indicator. The results were expressed in mg of acetic acid per 1 L of 100% *v*/*v* alcohol. The analysis of the volatile compounds in the obtained distillates was performed using a GC apparatus (Agilent 7890A; Agilent Technologies, Santa Clara, CA, USA) with a mass spectrometer (Agilent MSD 5975C; Agilent Technologies, Santa Clara, CA, USA) under the conditions described in the study of Pielech-Przybylska et al. [24]. Results were expressed as mg/L of 100% *v*/*v* alcohol.

Fermentation efficiency was calculated according to the stoichiometric Gay–Lussac equation in relation to total sugars (calculated as glucose) and expressed as a percentage (%) of the theoretical yield.

All experiments were performed in triplicate. The results of the chemical composition of the tested rye grains, the sweet and fermented mashes, the fermentation results (ethanol concentration, fermentation efficiency), the microbial analysis of mashes, as well as the results of the gas chromatography analysis of the obtained distillates were analysed using ANOVA with Tukey’s post hoc test with a significance level of *p* < 0.05. Moreover, principal component analysis (PCA) and hierarchical cluster analysis (HCA) were performed to classify the distillate samples and link volatile compounds with rye cultivation. All analyses were carried out using XLSTAT software (version 2024.3, Lumivero, Denver, CO, USA).

## 4. Conclusions

In this study, we compared the fermentation results and chemical composition of spirit distillates obtained from rye grain (Dańkowskie Złote cv.) cultivated conventionally and organically. Moreover, an effect of methods of starch liberation, saccharification and fermentation, as well as yeast strains were assessed.

The chemical composition of the tested rye grains remained unaffected by the conditions of rye cultivation; however, an effect on fermentation efficiency was observed. The processing of grain from conventional cultivation by the PLS method in conjunction with both the SSF and SHF strategies resulted in higher process efficiency than analogous samples from organic rye grain. No advantage of separate hydrolysis and fermentation (SHF) over simultaneous saccharification and fermentation (SSF) was found. The supplementation of mashes from both conventional and organic rye grain with protease had a positive effect on ethanol biosynthesis and fermentation efficiency. The chemical composition of the rye distillates obtained was found to be influenced to differing extents by the rye cultivation method and the use of methods of raw material processing, supportive proteolytic enzyme preparation, and yeast strains. The distillates obtained from conventionally cultivated rye were found to contain higher concentrations of carbonyl compounds, higher alcohols, and acetate esters compared with those from organic rye. The majority of distillates obtained from the rye mashes digested by this supportive enzyme, contained lower concentrations of the isoamyl alcohols (i.e., 2-methyl-butanol and 3-methyl-butanol) and higher concentrations of acetate esters of these alcohols. Importantly, all distillates obtained contained acetaldehyde within the recommended limit (<100 mg/L of 100% *v*/*v* alcohol) for distillates of agricultural origin in Polish distilleries. Moreover, methanol concentrations in rye distillates from both conventional and organic grain were significantly lower than the limit specified by EU Regulation No. 2019/787 for ethyl alcohol of agricultural origin (≤30 g/hL of 100% *v*/*v* alcohol).

Given the growing interest in organic products, organic rye grain could be considered a promising raw material for micro-distilleries. This is due to its satisfactory fermentation performance and the distinct chemical composition of the resulting distillates, which could give rise to unique sensory properties that warrant further study.

Our findings demonstrate that organic rye can deliver satisfactory process efficiency and generate distillates with a unique chemical profile. This supports the potential of organic raw materials as a viable alternative for the production of environmentally responsible spirits. These results are in line with the European Union’s broader sustainability objectives, which include reducing chemical inputs, preserving biodiversity and establishing low-impact agricultural value chains. However, it should be noted that these findings are based on a single variety of rye. Therefore, the conclusions presented may not be generalised to all rye genotypes, and further studies involving varieties with different genetic backgrounds are required to confirm the broader applicability of the observed trends.

## Figures and Tables

**Table 1:** Results of chemical analysis of tested rye grain (mean values, n = 3, see Supplementary Data for standard deviation (SD)).

Rye Cultivation	Moisture [%]	Protein (N × 6.25) [% d.m.]	Reducing Sugars [as g Glucose/100 g]	Starch [g/100 g]
Conventional	13.35 ^a^	11.46 ^a^	5.12 ^b^	48.74 ^a^
Organic	11.60 ^b^	10.93 ^a^	5.33 ^a^	50.45 ^a^

^a,b^—the differences between means in each column marked with different letters are statistically significant (one-way ANOVA with post hoc Tukey test, *p* < 0.05).

**Table 2:** Results of physico-chemical analysis of sweet rye mashes (mean values, n = 3, see Supplementary Data for standard deviation (SD)).

Rye Cultivation	Method of Starch Liberation	Method of Enzymatic Hydrolysis and Fermentation	Extract [°Blg]	Concentration [g/L]			
				Glucose	Maltose	Maltotriose	Dextrins
Conventional	PLS	SSF, amylolytic enzymes	16.35 ^f^	43.15 ^g^	17.02 ^e^	1.14 ^bcd^	82.05 ^a^
	PLS	SSF, amylolytic enzymes & protease	16.36 ^ef^	43.07 ^g^	17.13 ^e^	1.06 ^bcd^	82.24 ^a^
	PLS	SHF, amylolytic enzymes	16.53 ^d^	59.17 ^e^	25.43 ^bc^	0.83 ^de^	60.32 ^c^
	PLS	SHF, amylolytic enzymes & protease	16.49 ^de^	58.53 ^e^	25.14 ^c^	0.92 ^cde^	60.74 ^c^
	TP	SSF, amylolytic enzymes	16.45 ^def^	95.63 ^a^	2.44 ^h^	n.d.	53.26 ^e^
	TP	SSF, amylolytic enzymes & protease	16.42 ^def^	95.36 ^a^	2.27 ^h^	0.33 ^f^	53.54 ^e^
	TP	SHF, amylolytic enzymes	16.75 ^c^	92.35 ^b^	1.42 ^h^	n.d.	49.52 ^gh^
	TP	SHF, amylolytic enzymes & protease	16.75 ^c^	92.64 ^b^	1.46 ^h^	n.d.	49.34 ^h^
Organic	PLS	SSF, amylolytic enzymes	16.75 ^c^	44.95 ^f^	23.84 ^d^	1.42 ^ab^	74.93 ^b^
	PLS	SSF, amylolytic enzymes & protease	16.73 ^c^	45.13 ^f^	23.86 ^d^	1.45 ^abc^	75.86 ^b^
	PLS	SHF, amylolytic enzymes	16.94 ^b^	59.33 ^e^	26.82 ^a^	1.73 ^a^	48.53 ^h^
	PLS	SHF, amylolytic enzymes & protease	16.89 ^b^	59.22 ^e^	26.43 ^ab^	1.62 ^a^	48.13 ^h^
	TP	SSF, amylolytic enzymes	17.16 ^a^	73.04 ^d^	6.05 ^f^	n.d.	58.05 ^d^
	TP	SSF, amylolytic enzymes & protease	17.21 ^a^	73.42 ^d^	5.93 ^f^	0.52 ^ef^	58.16 ^d^
	TP	SHF, amylolytic enzymes	17.28 ^a^	80.91 ^c^	4.03 ^g^	0.24 ^f^	51.26 ^fg^
	TP	SHF, amylolytic enzymes & protease	17.21 ^a^	81.74 ^c^	4.42 ^g^	0.22 ^f^	51.35 ^f^

^a–h^—difference between means in each column marked with different letters are statistically significant (three-way ANOVA with post hoc Tukey test, *p* < 0.05); n.d.—not detected; PLS—pressureless starch liberation; TP—thermal-pressure starch liberation; SSF—simultaneous saccharification and fermentation; SHF—separate hydrolysis and fermentation.

**Table 3:** Chemical composition of fermented mashes (mean values, n = 3, see Supplementary Data for standard deviation (SD).

Rye Cultivation	Method of Starch Liberation	Method of Starch Saccharification and Fermentation	pH	Extract [°Blg]	Concentration [g/L]						
					Glucose	Maltose	Maltotriose	Dextrins	Glycerol	Lactic Acid	Acetic Acid
Conventional	PLS	SSF_A_SSHG1	4.28 ^bc^	0.66 ^cdef^	0.16 ^ghi^	0.88 ^b^	0.70 ^bc^	3.97 ^abc^	0.47 ^fgh^	0.01 ^j^	0.02 ^hi^
	PLS	SSF_A + P_SSHG1	4.12 ^defg^	0.63 ^efgh^	2.48 ^bcd^	0.24 ^b^	0.03 ^d^	3.77 ^abcd^	0.39 ^hij^	0.01 ^j^	0.01 ^hi^
	PLS	SSF_A_PDY(S)	4.29 ^bc^	0.65 ^defg^	0.05 ^i^	0.72 ^b^	0.64 ^c^	1.63 ^fgh^	0.53 ^efg^	0.01 ^j^	0.01 ^i^
	PLS	SSF_A + P_PDY(S)	4.04 ^fgh^	0.59 ^ghijk^	0.08 ^hi^	0.65 ^b^	0.81 ^a^	1.30 ^gh^	0.47 ^fgh^	0.01 ^j^	0.01 ^hi^
	PLS	SHF_A_SSHG1	4.29 ^bc^	0.69 ^cde^	2.32 ^bcdef^	2.00 ^ab^	0.03 ^d^	1.37 ^gh^	0.33 ^jkl^	0.02 ^hij^	0.01 ^i^
	PLS	SHF_A + P_SSHG1	4.07 ^fgh^	0.65 ^defg^	2.50 ^bcd^	0.60 ^b^	0.03 ^d^	3.20 ^bcde^	0.41 ^ghij^	0.02 ^hij^	0.01 ^i^
	PLS	SHF_A_PDY(S)	4.30 ^bc^	0.66 ^cdef^	0.06 ^i^	0.55 ^b^	0.74 ^ab^	4.60 ^ab^	0.46 ^fghi^	0.01 ^ij^	0.01 ^i^
	PLS	SHF_A + P_PDY(S)	4.08 ^efgh^	0.60 ^fghij^	0.33 ^efghi^	0.92 ^b^	0.70 ^bc^	4.77 ^a^	0.56 ^ef^	0.01 ^j^	0.01 ^i^
	TP	SSF_A_SSHG1	4.14 ^def^	0.56 ^ijklm^	1.28 ^defghi^	0.21 ^b^	0.06 ^d^	3.20 ^bcde^	0.52 ^efg^	0.04 ^ghij^	0.03 ^hi^
	TP	SSF_A + P_SSHG1	4.21 ^cd^	0.56 ^ijklm^	0.05 ^i^	0.21 ^b^	0.07 ^d^	3.77 ^abcd^	0.64 ^e^	0.01 ^j^	0.04 ^gh^
	TP	SSF_A_PDY(S)	4.19 ^cde^	0.58 ^hijkl^	0.04 ^i^	0.03 ^b^	0.01 ^d^	4.90 ^a^	0.45 ^fghij^	0.06 ^ghi^	0.06 ^fg^
	TP	SSF_A + P_PDY(S)	4.22 ^cd^	0.58 ^hijk^	0.05 ^i^	0.04 ^b^	0.02 ^d^	2.93 ^cdef^	0.57 ^ef^	0.07 ^g^	0.07 ^fg^
	TP	SHF_A_SSHG1	4.15 ^def^	0.68 ^cde^	0.08 ^hi^	0.24 ^b^	0.01 ^d^	2.93 ^cdef^	0.49 ^fgh^	0.03 ^ghij^	0.03 ^hi^
	TP	SHF_A + P_SSHG1	4.39 ^ab^	0.65 ^defg^	0.05 ^i^	0.16 ^b^	0.03 ^d^	0.93 ^gh^	0.43 ^ghij^	0.03 ^ghij^	0.02 ^hi^
	TP	SHF_A_PDY(S)	4.14 ^def^	0.65 ^defg^	0.06 ^i^	0.18 ^b^	0.03 ^d^	2.27 ^defg^	0.62 ^e^	0.04 ^ghij^	0.01 ^i^
	TP	SHF_A + P_PDY(S)	4.43 ^a^	0.61 ^fghi^	1.79 ^cdefghi^	0. 30 ^b^	0.02 ^d^	2.40 ^defg^	0.57 ^ef^	0.03 ^ghij^	0.01 ^i^
Organic	PLS	SSF_A_SSHG1	3.64 ^ij^	1.00 ^a^	3.61 ^bc^	1.55 ^ab^	0.03 ^d^	0.97 ^gh^	1. 46 ^cd^	0.34 ^f^	0.14 ^de^
	PLS	SSF_A + P_SSHG1	3.37 ^k^	0.96 ^ab^	6.25 ^a^	2.33 ^ab^	0.03 ^d^	0.34 ^h^	0.94 ^d^	0.42 ^e^	0.16 ^d^
	PLS	SSF_A_PDY(S)	3.58 ^j^	0.94 ^ab^	1.39 ^defghi^	1.23 ^b^	0.02 ^d^	1.20 ^gh^	1.46 ^a^	0.83 ^a^	0.35 ^a^
	PLS	SSF_A + P_PDY(S)	3.37 ^k^	0.94 ^b^	2.18 ^bcdefgh^	1.13 ^b^	0.03 ^d^	1.27 ^gh^	1.20 ^b^	0.79 ^a^	0.22 ^c^
	PLS	SHF_A_SSHG1	3.71 ^i^	0.69 ^cde^	2.37 ^bcde^	0.50 ^b^	0.02 ^d^	2.37 ^defg^	1.02 ^cd^	0.44 ^de^	0.07 ^f^
	PLS	SHF_A + P_SSHG1	3.61 ^ij^	0.70 ^cd^	4.16 ^ab^	2.22 ^ab^	0.02 ^d^	1.53 ^fgh^	1.07 ^c^	0.61 ^c^	0.16 ^d^
	PLS	SHF_A_PDY(S)	3.66 ^ij^	0.69 ^cde^	0.07 ^i^	1.33 ^b^	0.03 ^d^	1.17 ^gh^	1.27 ^b^	0.71 ^b^	0.27 ^b^
	PLS	SHF_A + P_PDY(S)	3.61 ^ij^	0.72 ^c^	0.25 ^fghi^	4.97 ^a^	0.03 ^d^	1.28 ^gh^	1.20 ^b^	0.71 ^b^	0.20 ^c^
	TP	SSF_A_SSHG1	4.01 ^gh^	0.54 ^ijklm^	0.30 ^efghi^	0.34 ^b^	0.03 ^d^	1.43 ^fgh^	0.41 ^ghij^	0.49 ^d^	0.11 ^e^
	TP	SSF_A + P_SSHG1	4.00 ^gh^	0.52 ^lm^	1.12 ^defghi^	0.48 ^b^	0.02 ^d^	0. 91 ^gh^	0.22 ^klm^	0.07 ^gh^	0.01 ^hi^
	TP	SSF_A_PDY(S)	3.97 ^h^	0.54 ^jklm^	0.30 ^efghi^	0.42 ^b^	0.03 ^d^	1.08 ^gh^	0.22 ^lm^	0.02 ^ghij^	0.01 ^hi^
	TP	SSF_A + P_PDY(S)	4.01 ^gh^	0.53 ^klm^	0.86 ^defghi^	0.33 ^b^	0.01 ^d^	1.20 ^gh^	0.20 ^m^	0.03 ^ghij^	0.03 ^hi^
	TP	SHF_A_SSHG1	4.01 ^gh^	0.53 ^klm^	0.50 ^defghi^	0.70 ^b^	0.02 ^d^	0.61 ^h^	0.20 ^m^	0.04 ^ghij^	0.01 ^i^
	TP	SHF_A + P_SSHG1	4.00 ^gh^	0.50 ^m^	0.14 ^ghi^	0.43 ^b^	0.01 ^d^	1.19 ^gh^	0.34 ^ijk^	0.04 ^ghij^	0.01 ^i^
	TP	SHF_A_PDY(S)	3.98 ^h^	0.51 ^m^	2.23 ^bcdefg^	0.22 ^b^	0.01 ^d^	1.80 ^efgh^	0.21 ^lm^	0.05 ^ghij^	0.01 ^i^
	TP	SHF_A + P_PDY(S)	4.01 ^gh^	0.50 ^m^	0.30 ^efghi^	0.26 ^b^	0.02 ^d^	1.73 ^efgh^	0.23 ^klm^	0.03 ^ghij^	0.01 ^i^

^a–m^—difference between means in each column marked with different letters are statistically significant (three-way ANOVA with post hoc Tukey test, *p* < 0.05); PLS—pressureless starch liberation; TP—thermal-pressure starch liberation; SSF—simultaneous saccharification and fermentation; SHF—separate hydrolysis and fermentation; A—amylolytic enzymes; A + P—amylolytic enzymes + protease; SSHG1—SafSpirit HG-1 yeast; PDY(S)—Pinnacle Distillers Yeast (S).

**Table 4:** Fermentation results of rye mashes (mean values, n = 3, see Supplementary Data for standard deviation (SD)).

Rye Cultivation	Method of Starch Liberation	Method of Starch Saccharification and Fermentation	Ethanol [% *v*/*v*]	Fermentation Efficiency [% of Theoretical]
Conventional	PLS	SSF_A_SSHG1	8.66 ^cdef^	87.31 ^efghijk^
	PLS	SSF_A + P_SSHG1	9.13 ^a^	91.99 ^abcd^
	PLS	SSF_A_PDY(S)	8.66 ^cdef^	87.31 ^efghijk^
	PLS	SSF_A + P_PDY(S)	8.96 ^abc^	90.29 ^abcdefg^
	PLS	SHF_A_SSHG1	8.98 ^abc^	90.23 ^abcdefg^
	PLS	SHF_A + P_SSHG1	9.25 ^a^	92.99 ^a^
	PLS	SHF_A_PDY(S)	9.21 ^a^	92.57 ^ab^
	PLS	SHF_A + P_PDY(S)	9.13 ^a^	91.72 ^abcd^
	TP	SSF_A_SSHG1	8.45 ^defgh^	82.96 ^lmn^
	TP	SSF_A + P_SSHG1	9.04 ^ab^	88.76 ^cdefghij^
	TP	SSF_A_PDY(S)	8.28 ^ghij^	81.30 ^mn^
	TP	SSF_A + P_PDY(S)	8.66 ^cdef^	85.03 ^jklm^
	TP	SHF_A_SSHG1	8.32 ^fghi^	86.34 ^hijkl^
	TP	SHF_A + P_SSHG1	8.75 ^bcd^	90.72 ^abcdef^
	TP	SHF_A_PDY(S)	8.07 ^ijkl^	83.71 ^klm^
	TP	SHF_A + P_PDY(S)	8.66 ^cdef^	89.85 ^abcdefgh^
Organic	PLS	SSF_A_SSHG1	7.52 ^n^	75.05 ^o^
	PLS	SSF_A + P_SSHG1	8.15 ^hijk^	81.37 ^mn^
	PLS	SSF_A_PDY(S)	7.94 ^jklm^	79.27 ^n^
	PLS	SSF_A + P_PDY(S)	8.70 ^bcde^	86.86 ^ghijk^
	PLS	SHF_A_SSHG1	7.77 ^lmn^	83.85 ^klm^
	PLS	SHF_A + P_SSHG1	8.20 ^hijk^	88.41 ^defghij^
	PLS	SHF_A_PDY(S)	8.07 ^ijkl^	87.04 ^fghijk^
	PLS	SHF_A + P_PDY(S)	8.58 ^defg^	92.51 ^abc^
	TP	SSF_A_SSHG1	7.86 ^klmn^	84.37 ^klm^
	TP	SSF_A + P_SSHG1	8.28 ^ghij^	88.90 ^bcdefghi^
	TP	SSF_A_PDY(S)	7.73 ^lmn^	83.01 ^lmn^
	TP	SSF_A + P_PDY(S)	8.28 ^ghij^	88.90 ^bcdefghi^
	TP	SHF_A_SSHG1	7.86 ^klmn^	85.46 ^ijkl^
	TP	SHF_A + P_SSHG1	8.37 ^efghi^	90.97 ^abcde^
	TP	SHF_A_PDY(S)	7.69 ^mn^	83.62 ^klm^
	TP	SHF_A + P_PDY(S)	8.20 ^hijk^	89.13 ^bcdefghi^

^a–o^—difference between means in each column marked with different letters are statistically significant (three-way ANOVA with post hoc Tukey test, *p* < 0.05); PLS—pressureless starch liberation; TP—thermal-pressure starch liberation; SSF—simultaneous saccharification and fermentation; SHF—separate hydrolysis and fermentation; A—amylolytic enzymes; A + P—amylolytic enzymes + protease; SSHG1—SafSpirit HG-1 yeast; PDY(S)—Pinnacle Distillers Yeast (S).

**Table 5:** Acidity and carbonyl compounds in tested rye distillates (mean values, n = 3, see Supplementary Data for standard deviation (SD)).

Rye Cultivation	Method of Starch Liberation	Method of Starch Saccharification and Fermentation	Acidity as Acetic Acid	Acetone	Acetaldehyde	2,3-Butanedione	3-Methylbutanal	2-Methylbutanal	Phenylacetaldehyde
			Concentration [mg/L of 100% *v*/*v* Alcohol]						
Conventional	PLS	SSF_A_SSHG1	115.22 ^cdefg^	4.55 ^ghij^	32.13 ^abcdefg^	3.45 ^f^	0.89 ^fgh^	1.479 ^efg^	1.42 ^ghi^
	PLS	SSF_A + P_SSHG1	122.13 ^bcdefg^	12.49 ^cd^	31.54 ^abcdefg^	2.29 ^f^	1.62 ^e^	1.08 ^hij^	1.34 ^ghij^
	PLS	SSF_A_PDY(S)	112.32 ^defg^	13.80 ^bc^	28.75 ^abcdefgh^	3.07 ^f^	0.67 ^fghijk^	1.29 ^fgh^	1.50 ^g^
	PLS	SSF_A + P_PDY(S)	125.14 ^bcdefg^	15.58 ^b^	32.66 ^abcde^	2.62 ^f^	1.05 ^f^	0.67 ^klmn^	1.20 ^ghijkl^
	PLS	SHF_A_SSHG1	156.42 ^a^	9.53 ^e^	32.13 ^abcdefg^	2.80 ^f^	0.62 ^fghijk^	1.10 ^ghij^	1.47 ^gh^
	PLS	SHF_A + P_SSHG1	121.11 ^bcdefg^	12.57 ^cd^	34.07 ^ab^	2.56 ^f^	0.82 ^fghij^	0.55 ^lmno^	1.22 ^ghijk^
	PLS	SHF_A_PDY(S)	108.43 ^fg^	1.17 ^mno^	26.82 ^cdefgh^	2.58 ^f^	0.66 ^fghijk^	0.98 ^hijk^	1.52 ^g^
	PLS	SHF_A + P_PDY(S)	123.33 ^bcdefg^	9.497 ^e^	25.57 ^efgh^	2.61 ^f^	0.85 ^fghi^	0.55 ^lmno^	1.15 ^ghijklm^
	TP	SSF_A_SSHG1	106.24 ^g^	11.54 ^de^	29.31 ^abcdefgh^	5.26 ^ef^	1.98 ^e^	1.17 ^ghij^	2.89 ^f^
	TP	SSF_A + P_SSHG1	112.42 ^defg^	2.08 ^klmno^	25.38 ^fgh^	6.22 ^ef^	7.28 ^a^	3.19 ^a^	6.58 ^a^
	TP	SSF_A_PDY(S)	110.51 ^efg^	30.27 ^a^	30.38 ^abcdefg^	44.68 ^c^	3.96 ^cd^	2.45 ^c^	4.14 ^cd^
	TP	SSF_A + P_PDY(S)	112.33 ^defg^	1.17 ^mno^	31.54 ^abcdefg^	3.81 ^f^	1.75 ^e^	0.55 ^lmno^	4.86 ^b^
	TP	SHF_A_SSHG1	115.31 ^cdefg^	2.76 ^jklmn^	27.04 ^bcdefgh^	4.64 ^f^	3.40 ^d^	1.71 ^de^	3.22 ^ef^
	TP	SHF_A + P_SSHG1	126.34 ^bcdef^	3.27 ^ijklm^	25.21 ^gh^	33.81 ^d^	4.54 ^b^	1.93 ^d^	3.79 ^cde^
	TP	SHF_A_PDY(S)	119.32 ^bcdefg^	6.93 ^f^	28.58 ^abcdefgh^	6.49 ^ef^	3.96 ^cd^	2.56 ^bc^	4.35 ^bc^
	TP	SHF_A + P_PDY(S)	127.22 ^bcde^	4.01 ^hijk^	34.18 ^ab^	32.06 ^d^	4.19 ^bc^	1.55 ^ef^	3.69 ^de^
Organic	PLS	SSF_A_SSHG1	122.41 ^bcdefg^	0.69 ^no^	33.41 ^abcd^	3.08 ^f^	0.29 ^ijkl^	0.85 ^ijklm^	0.59 ^mno^
	PLS	SSF_A + P_SSHG1	127.33 ^bcde^	1.47 ^lmno^	22.18 ^h^	1.33 ^f^	0.44 ^ghijkl^	2.50 ^c^	0.64 ^klmno^
	PLS	SSF_A_PDY(S)	122.42 ^bcdefg^	0.56 ^o^	28.82 ^abcdefgh^	1.74 ^f^	n.d.	0.51 ^mno^	0.61 ^lmno^
	PLS	SSF_A + P_PDY(S)	129.31 ^bcd^	1.77 ^lmno^	22.25 ^h^	1.41 ^f^	0.57 ^fghijk^	2.90 ^ab^	0.71 ^klmno^
	PLS	SHF_A_SSHG1	114.30 ^cdefg^	0.55 ^o^	32.78 ^abcde^	3.58 ^f^	0.28 ^jkl^	1.07 ^hij^	0.46 ^o^
	PLS	SHF_A + P_SSHG1	125.32 ^bcdef^	6.05 ^fgh^	25.04 ^gh^	1.93 ^f^	0.54 ^fghijkl^	2.71 ^bc^	0.76 ^jklmno^
	PLS	SHF_A_PDY(S)	116.24 ^cdefg^	1.02 ^no^	33.76 ^abc^	4.14 ^f^	0.35 ^hijkl^	0.92 ^hijkl^	0.51 ^no^
	PLS	SHF_A + P_PDY(S)	128.61 ^bcde^	1.20 ^mno^	28.09 ^abcdefgh^	1.61 ^f^	0.57 ^fghijk^	2.35 ^c^	0.85 ^ijklmno^
	TP	SSF_A_SSHG1	115.21 ^cdefg^	3.47 ^ijkl^	34.93 ^a^	5.99 ^ef^	0.48 ^ghijkl^	0.94 ^hijk^	1.14 ^ghijklm^
	TP	SSF_A + P_SSHG1	124.20 ^bcdefg^	4.36 ^ghij^	31.63 ^abcdefg^	47.42 ^c^	0.37 ^hijkl^	0.25 ^o^	1.05 ^ghijklmno^
	TP	SSF_A_PDY(S)	116.33 ^cdefg^	6.20 ^fg^	28.67 ^abcdefgh^	6.80 ^ef^	0.32 ^ijkl^	1.19 ^fghi^	1.32 ^ghij^
	TP	SSF_A + P_PDY(S)	131.41 ^bc^	0.16 ^o^	26.189 ^defgh^	10.23 ^e^	0.25 ^kl^	0.41 ^no^	0.81 ^jklmno^
	TP	SHF_A_SSHG1	123.15 ^bcdefg^	5.07 ^fghi^	33.33 ^abcd^	3.79 ^f^	0.30 ^ijkl^	0.79 ^jklm^	0.98 ^ghijklmno^
	TP	SHF_A + P_SSHG1	130.33 ^bcd^	1.85 ^klmno^	32.47 ^abcdef^	54.19 ^b^	0.24 ^kl^	0.48 ^mno^	0.89 ^hijklmno^
	TP	SHF_A_PDY(S)	125.22 ^bcdefg^	10.24 ^e^	28.87 ^abcdefgh^	4.78 ^ef^	0.39 ^hijkl^	0.85 ^ijklm^	1.09 ^ghijklmn^
	TP	SHF_A + P_PDY(S)	135.42 ^b^	n.d.	29.94 ^abcdefg^	87.64 ^a^	0.98 ^fg^	1.17 ^ghi^	0.69 ^klmno^

^a–o^—difference between means in each column marked with different letters are statistically significant (three-way ANOVA with post hoc Tukey test, *p* < 0.05); n.d.—not detected; PLS—pressureless starch liberation; TP—thermal-pressure starch liberation; SSF—simultaneous saccharification and fermentation; SHF—separate hydrolysis and fermentation; A—amylolytic enzymes; A + P—amylolytic enzymes + protease; SSHG1—SafSpirit HG-1 yeast; PDY(S)—Pinnacle Distillers Yeast (S).

**Table 6:** Methanol and higher alcohols in tested rye distillates (mean values, n = 3, see Supplementary Data for standard deviations (SD)).

Rye Cultivation	Method of Starch Liberation	Method of Starch Saccharification and Fermentation	Methanol	2-Propanol	1-Propanol	2-Methylbutanol and 3-Methylbutanol	1-Butanol
			Concentration [mg/L of 100% *v*/*v* Alcohol]				
Conventional	PLS	SSF_A_SSHG1	54.70 ^fghi^	0.48 ^r^	268.04 ^jkl^	3150.31 ^bc^	11.89 ^bcdef^
	PLS	SSF_A + P_SSHG1	46.17 ^ij^	2.98 ^gh^	259.40 ^kl^	1415.50 ^ef^	11.23 ^cdef^
	PLS	SSF_A_PDY(S)	50.16 ^ghi^	1.78 ^klmn^	217.63 ^l^	2976.96 ^bc^	11.37 ^bcdef^
	PLS	SSF_A + P_PDY(S)	47.87 ^hi^	2.46 ^hijkl^	382.64 ^defgh^	1495.21 ^ef^	11.68 ^bcdef^
	PLS	SHF_A_SSHG1	50.97 ^ghi^	0.81 ^pqr^	230.14 ^kl^	3300.92 ^ab^	11.27 ^cdef^
	PLS	SHF_A + P_SSHG1	48.03 ^ij^	1.77 ^klmno^	513.17 ^b^	2365.05 ^d^	12.43 ^bcde^
	PLS	SHF_A_PDY(S)	50.00 ^ghi^	0.97 ^opqr^	228.91 ^kl^	3171.46 ^bc^	11.30 ^bcdef^
	PLS	SHF_A + P_PDY(S)	46.09 ^ij^	1.73 ^lmno^	509.98 ^b^	2366.05 ^d^	10.46 ^ef^
	TP	SSF_A_SSHG1	66.64 ^ef^	2.75 ^ghi^	237.64 ^kl^	2406.37 ^d^	14.08 ^b^
	TP	SSF_A + P_SSHG1	63.08 ^efgh^	8.44 ^a^	463.16 ^bcd^	1574.76 ^ef^	14.08 ^b^
	TP	SSF_A_PDY(S)	90.18 ^bcd^	2.41 ^gh^	382.64 ^defgh^	3727.79 ^a^	14.08 ^b^
	TP	SSF_A + P_PDY(S)	59.39 ^efghi^	4.60 ^de^	256.97 ^kl^	832.59 ^ghi^	10.60 ^def^
	TP	SHF_A_SSHG1	59.25 ^efghi^	1.98 ^ijklmn^	265.48 ^kl^	2357.42 ^d^	13.73 ^bc^
	TP	SHF_A + P_SSHG1	63.84 ^efg^	2.83 ^hijk^	453.57^bcde^	1432.54 ^ef^	13.42 ^bc^
	TP	SHF_A_PDY(S)	63.46 ^efg^	1.40 ^nopq^	300.97 ^hijkl^	2414.74 ^d^	13.88 ^bc^
	TP	SHF_A + P_PDY(S)	64.93 ^efg^	2.56 ^hijk^	485.89 ^bc^	1485.87 ^ef^	n.d.
Organic	PLS	SSF_A_SSHG1	59.83 ^efgh^	4.87 ^cde^	261.95 ^kl^	701.20 ^hij^	12.09 ^bcdef^
	PLS	SSF_A + P_SSHG1	46.24 ^ij^	8.32 ^a^	366.32 ^efghi^	696.70 ^hij^	9.67 ^efg^
	PLS	SSF_A_PDY(S)	74.96 ^de^	3.38 ^fg^	111.98 ^m^	688.85 ^hij^	n.d.
	PLS	SSF_A + P_PDY(S)	45.55 ^ij^	5.58 ^bc^	273.85 ^jkl^	718.87 ^hij^	n.d.
	PLS	SHF_A_SSHG1	45.20 ^ij^	1.52 ^mnop^	313.88 ^ghijk^	812.62 ^hi^	n.d.
	PLS	SHF_A + P_SSHG1	44.20 ^ij^	4.06 ^ef^	357.93 ^fghij^	782.74 ^hij^	9.62 ^fg^
	PLS	SHF_A_PDY(S)	62.59 ^efgh^	0.61^qr^	219.52 ^l^	612.26 ^ij^	7.19 ^g^
	PLS	SHF_A + P_PDY(S)	62.44 ^efgh^	5.18 ^cd^	401.06 ^cdefg^	1136.79 ^fgh^	11.40 ^bcdef^
	TP	SSF_A_SSHG1	98.26 ^ab^	2.60 ^ghij^	245.06 ^kl^	2427.99 ^d^	n.d.
	TP	SSF_A + P_SSHG1	92.04 ^bc^	4.43 ^cde^	397.62 ^cdefg^	1369.88 ^ef^	n.d.
	TP	SSF_A_PDY(S)	100.70 ^ab^	2.24 ^hijklm^	292.06 ^ijkl^	2444.36 ^d^	13.76 ^bc^
	TP	SSF_A + P_PDY(S)	99.39 ^ab^	6.10 ^b^	407.39 ^cdef^	1330.59 ^efg^	11.72 ^bcdef^
	TP	SHF_A_SSHG1	82.63 ^cd^	1.84 ^jklmn^	281.28 ^ijkl^	2431.50 ^d^	18.03 ^a^
	TP	SHF_A + P_SSHG1	101.85 ^ab^	2.69 ^ghi^	540.19 ^b^	1777.34 ^e^	12.44 ^bcde^
	TP	SHF_A_PDY(S)	89.93 ^bcd^	1.31 ^nopq^	367.38 ^efghi^	2660.34 ^cd^	13.35 ^bcd^
	TP	SHF_A + P_PDY(S)	112.64 ^a^	2.78 ^ghi^	836.45 ^a^	2700.25 ^cd^	18.86 ^a^

^a–r^—differences between means in each column marked with different letters are statistically significant (three-way ANOVA with post hoc Tukey test, *p* < 0.05), n.d.—not detected; PLS—pressureless starch liberation; TP—thermal-pressure starch liberation; SSF—simultaneous saccharification and fermentation; SHF—separate hydrolysis and fermentation; A—amylolytic enzymes; A + P—amylolytic enzymes + protease; SSHG1—SafSpirit HG-1 yeast; PDY(S)—Pinnacle Distillers Yeast (S).

**Table 7:** Esters in tested rye distillates (mean values, n = 3, see Supplementary Data for standard deviations (SD)).

Rye Cultivation	Method of Starch Liberation	Method of Starch Saccharification and Fermentation	Ethyl Acetate	Ethyl Butyrate	Isobutyl Acetate	3-Methylbutyl Acetate	2-Methylbutyl Acetate	Ethyl Hexanoate	Ethyl Heptanoate	Ethyl Octanoate	Ethyl Decanoate	Ethyl Tetradecanoate
			Concentration [mg/L of 100% *v*/*v* Alcohol]									
Conventional	PLS	SSF_A_SSHG1	186.65 ^b^	0.14 ^jklm^	0.24 ^efg^	4.31 ^bc^	0.49 ^c^	1.33 ^def^	0.11 ^ef^	5.19 ^efg^	7.48 ^fgh^	2.41 ^c^
	PLS	SSF_A + P_SSHG1	143.32 ^gh^	0.18 ^ghijk^	0.38 ^b^	5.49 ^a^	0.77 ^ab^	1.81 ^c^	0.15 ^d^	8.48 ^c^	9.75 ^de^	1.52 ^defgh^
	PLS	SSF_A_PDY(S)	168.53 ^de^	0.13 ^klm^	0.18 ^hi^	3.52 ^de^	0.42 ^cde^	1.28 ^defg^	0.14 ^d^	5.87 ^ef^	5.52 ^hijk^	1.30 ^efghij^
	PLS	SSF_A + P_PDY(S)	140.32 ^ghi^	0.21 ^efgh^	0.38 ^b^	5.49 ^a^	0.78 ^ab^	1.57 ^cde^	0.13 ^de^	5.19 ^efg^	10.37 ^d^	3.90 ^a^
	PLS	SHF_A_SSHG1	179.54 ^bcd^	0.15 ^ijkl^	0.31 ^cd^	3.95 ^bcd^	0.45 ^cd^	1.25 ^efghi^	0.09 ^fg^	4.52 ^fghij^	5.46 ^ijk^	1.55 ^defgh^
	PLS	SHF_A + P_SSHG1	152.33 ^fg^	0.19 ^ghij^	0.41 ^ab^	5.63 ^a^	0.81 ^ab^	1.29 ^defg^	0.09 ^fg^	4.21 ^ghijk^	4.46 ^klm^	1.52^defgh^
	PLS	SHF_A_PDY(S)	184.62 ^bc^	0.15 ^ijkl^	0.29 ^de^	3.71 ^cde^	0.44 ^cd^	1.23 ^efghij^	0.11 ^ef^	4.67 ^fghi^	7.14 ^ghi^	1.92 ^d^
	PLS	SHF_A + P_PDY(S)	150.33 ^fg^	0.16 ^hijk^	0.36 ^bc^	4.54 ^b^	0.71 ^b^	0.98 ^ghijk^	0.08 ^gh^	3.09 ^jklmn^	5.39 ^ijk^	3.37 ^b^
	TP	SSF_A_SSHG1	130.22 ^hi^	0.10 ^lmn^	n.d.	0.61 ^mn^	0.07 ^kl^	0.32 ^no^	0.03 ^j^	2.16 ^mn^	1.89 ^n^	0.90 ^ijkl^
	TP	SSF_A + P_SSHG1	139.33 ^ghi^	0.20 ^fghi^	0.04 ^m^	1.10 ^jklmn^	0.16 ^ijk^	0.46 ^mn^	0.03 ^j^	2.63 ^lmn^	3.53 ^klmn^	1.61 ^defg^
	TP	SSF_A_PDY(S)	179.52 ^bcd^	0.29 ^cd^	0.15 ^ij^	0.07 ^o^	0.36 ^def^	1.52 ^cdef^	0.14 ^d^	6.40 ^de^	2.64 ^mn^	0.77 ^kl^
	TP	SSF_A + P_PDY(S)	109.15 ^j^	n.d.	n.d.	3.10 ^ef^	0.49 ^c^	0.09 ^o^	n.d.	0.11 ^o^	n.d.	0.46 ^l^
	TP	SHF_A_SSHG1	161.42 ^ef^	0.19 ^ghij^	0.08 ^lm^	1.50 ^hijkl^	0.17 ^ijk^	0.64 ^klmn^	0.04 ^ij^	4.27 ^ghijk^	4.82 ^jkl^	1.17 ^ghijk^
	TP	SHF_A + P_SSHG1	141.33 ^ghi^	0.26 ^cde^	0.09 ^klm^	1.72 ^hijk^	0.20 ^hij^	0.62 ^lmn^	0.04 ^ij^	4.23 ^ghijk^	8.10 ^efg^	2.76 ^c^
	TP	SHF_A_PDY(S)	177.52 ^bcd^	0.22 ^efg^	0.09 ^klm^	1.65 ^hijk^	0.21 ^hij^	0.84 ^kl^	0.08 ^gh^	4.96 ^efgh^	4.97 ^jkl^	1.48 ^defgh^
	TP	SHF_A + P_PDY(S)	152.35 ^fg^	0.25 ^cdef^	0.14 ^ijk^	1.87 ^hi^	0.23 ^ghij^	0.67 ^klm^	0.07 ^gh^	5.02 ^efgh^	7.25 ^fghi^	1.89 ^d^
Organic	PLS	SSF_A_SSHG1	127.05 ^i^	0.05 ^n^	0.15 ^ij^	0.65 ^mn^	0.15 ^ijk^	0.91 ^ijkl^	0.06 ^hi^	2.03 ^n^	2.00 ^n^	1.13 ^hijk^
	PLS	SSF_A + P_SSHG1	138.02 ^ghi^	0.09 ^mn^	0.36 ^bc^	0.83 ^lmn^	0.32 ^efg^	1.27 ^efgh^	0.09 ^fg^	3.59 ^hijklm^	4.33 ^klm^	1.56 ^defgh^
	PLS	SSF_A_PDY(S)	105.55 ^j^	n.d.	n.d.	n.d.	n.d.	0.04 ^o^	n.d.	0.08 ^o^	n.d.	0.440 ^l^
	PLS	SSF_A + P_PDY(S)	147.09 ^fg^	0.10 ^lmn^	0.18 ^hi^	0.68 ^mno^	0.22 ^ghij^	0.89 ^jkl^	0.07 ^gh^	2.09 ^n^	2.19 ^n^	0.90 ^ijkl^
	PLS	SHF_A_SSHG1	141.03 ^ghi^	0.13 ^klm^	0.22 ^fgh^	1.07 ^klmn^	0.29 ^fgh^	1.22 ^fghij^	0.06 ^hi^	2.56 ^lmn^	3.09 ^lmn^	1.28 ^efghij^
	PLS	SHF_A + P_SSHG1	179.05 ^bcd^	0.14 ^jklm^	0.40 ^ab^	1.34 ^ijklm^	0.49 ^c^	1.30 ^defg^	0.07 ^gh^	4.99 ^efgh^	7.87 ^efg^	1.74 ^de^
	PLS	SHF_A_PDY(S)	143.10 ^gh^	0.06 ^n^	n.d.	0.47 ^n^	0.16 ^ijk^	1.44^def^	0.13 ^de^	4.55 ^fghij^	3.68 ^klmn^	0.89 ^jkl^
	PLS	SHF_A + P_PDY(S)	176.02 ^bcde^	0.25 ^def^	0.22 ^fgh^	2.71 ^fg^	0.71 ^b^	3.82 ^a^	0.34 ^a^	13.57 ^b^	19.17^b^	2.85 ^c^
	TP	SSF_A_SSHG1	171.04 ^cde^	0.21 ^efgh^	0.100 ^jkl^	1.15 ^ijklmn^	0.17 ^ijk^	0.89 ^jkl^	0.06 ^hi^	2.99 ^klmn^	3.54 ^klmn^	1.25 ^fghij^
	TP	SSF_A + P_SSHG1	143.05 ^gh^	0.21 ^efgh^	0.12 ^jkl^	1.84 ^hi^	0.18 ^ij^	0.67 ^klm^	0.04 ^ij^	3.33 ^ijklmn^	4.59 ^jklm^	1.68 ^def^
	TP	SSF_A_PDY(S)	178.20 ^bcd^	0.19 ^ghij^	0.11 ^jkl^	0.99 ^klmn^	0.18 ^ij^	0.76 ^klm^	0.07 ^gh^	3.46 ^ijklmn^	3.36 ^lmn^	1.37 ^efghi^
	TP	SSF_A + P_PDY(S)	143.10 ^gh^	0.16 ^hijk^	0.09 ^klm^	1.82 ^hij^	0.14 ^jk^	0.61 ^lmn^	0.06 ^hi^	3.77 ^ghijkl^	9.19^def^	2.44^c^
	TP	SHF_A_SSHG1	189.00 ^b^	0.18 ^ghijk^	0.11 ^jkl^	2.10 ^gh^	0.25 ^ghi^	0.93 ^hijkl^	0.07 ^gh^	4.38 ^ghijk^	6.49 ^ghij^	1.55 ^defgh^
	TP	SHF_A + P_SSHG1	174.05 ^bcde^	0.39 ^b^	0.25 ^ef^	3.27 ^def^	0.43 ^cd^	2.21 ^b^	0.18 ^c^	14.52 ^b^	27.69 ^a^	4.21 ^a^
	TP	SHF_A_PDY(S)	211.10 ^a^	0.31 ^c^	0.19 ^ghi^	3.27 ^def^	0.37 ^def^	1.62 ^cd^	0.18 ^c^	7.53 ^cd^	7.64 ^fg^	1.52 ^defgh^
	TP	SHF_A + P_PDY(S)	223.05 ^a^	0.91 ^a^	0.45 ^a^	6.01 ^a^	0.84 ^a^	4.06 ^a^	0.31 ^b^	17.73 ^a^	14.58 ^c^	2.74 ^c^

^a–o^—differences between means in each column marked with different letters are statistically significant (three-way ANOVA with post hoc Tukey test, *p* < 0.05), n.d.—not detected; PLS—pressureless starch liberation; TP—thermal-pressure starch liberation; SSF—simultaneous saccharification and fermentation; SHF—separate hydrolysis and fermentation; A—amylolytic enzymes; A + P—amylolytic enzymes + protease; SSHG1—SafSpirit HG-1 yeast; PDY(S)—Pinnacle Distillers Yeast (S).

## Data Availability

The original contributions presented in this study are included in the article. Further inquiries can be directed to the corresponding author.

## Supplementary Materials

The following supporting information can be downloaded at: https://www.mdpi.com/article/10.3390/molecules31010157/s1, Table S1: Results of chemical analysis of tested rye grain (mean values with standard deviations (SD)); Table S2: Results of physico-chemical analysis of rye sweet mashes (mean values with standard deviations (SD)); Table S3: Results of physico-chemical analysis of rye fermented mashes (mean values with standard deviations (SD)); Table S3.1: Microbial analysis of rye mashes; Table S4: Fermentation results of rye mashes (mean values with standard deviations (SD)); Table S5: Acidity and carbonyl compounds in tested rye distillates (mean values with standard deviations (SD)); Table S6: Methanol and higher alcohols concentrations in tested rye distillates (mean values with standard deviations (SD)); Table S7: Esters in tested rye distillates (mean values with standard deviations (SD)); Table S8: Eigenvalues with % of total variance explained by principal components.
