# Supplementary material for: Impact of Conventional and Organic Cultivation Methods on Fermentation Efficiency and Volatile Composition of Rye Distillates
Source: Molecules. 2026 Jan 1;31(1):157. doi: 10.3390/molecules31010157 (PMC12787293; doi:10.3390/molecules31010157)
Supplement: Supplementary file 1 [file molecules-31-00157-s001.zip › Table S3.1. Microbial analysis of rye mashes.pdf]

Table S3.1. Microbial analysis of rye mashes.

Rye cultivation	Method of starch liberation	Method of starch saccharification and fermentation	Yeast Count log (cfu/mL)		Lactic Acid Bacteria (LAB) Count log (cfu/mL)		Total Mesophilic Bacteria (TMB) Count log (cfu/mL)	
			before fermentation	after fermentation	before fermentation	after fermentation	before fermentation	after fermentation
Conventional	PLS	SSF_A_SSHG1	6.85 ± 0.35 ^{aB}	7.95 ± 0.20 ^{aA}	<1.00 ^{bA}	1.18 ± 0.12^{cA}	1.18 ± 0.06 ^{bA}	1.60 ± 0.13 ^{cB}
	PLS	SSF_A+P_SSHG1	6.85 ± 0.40 ^{aB}	8.00 ± 0.22 ^{aA}	<1.00 ^{bA}	1.18 ± 0.12 ^{cA}	1.18 ± 0.06^{bA}	1.45 ± 0.20^{cA}
	PLS	SSF_A_PDY(S)	6.85 ± 0.25 ^{aB}	8.10 ± 0.15 ^{aA}	<1.00 ^{bA}	1.18 ± 0.12 ^{cA}	1.18 ± 0.06^{bA}	1.39 ± 0.11 ^{cA}
	PLS	SSF_A+P_PDY(S)	6.85 ± 0.40 ^{aB}	8.05 ± 0.22 ^{aA}	<1.00 ^{bA}	1.18 ± 0.12 ^{cA}	1.18 ± 0.06 ^{bA}	1.35 ± 0.22 ^{cA}
	PLS	SHF_A_SSHG1	6.85 ± 0.29 ^{aB}	8.08 ± 0.30 ^{aA}	<1.00 ^{bA}	1.18 ± 0.12 ^{cA}	1.18 ± 0.06 ^{bA}	1.45 ± 0.18 ^{cA}
	PLS	SHF_A+P_SSHG1	6.85 ± 0.40 ^{aB}	8.05 ± 0.12 ^{aA}	<1.00 ^{bA}	1.18 ± 0.12 ^{cA}	1.18 ± 0.06 ^{bA}	1.60 ± 0.15 ^{cA}
	PLS	SHF_A_PDY(S)	6.85 ± 0.40 ^{aB}	7.90 ± 0.32 ^{aA}	<1.00 ^{bA}	1.18 ± 0.12 ^{cA}	1.18 ± 0.06 ^{bA}	1.45 ± 0.20 ^{cA}
	PLS	SHF_A+P_PDY(S)	6.85 ± 0.32 ^{aB}	8.04 ± 0.22 ^{aA}	<1.00 ^{bA}	1.18 ± 0.12 ^{cA}	1.18 ± 0.06 ^{bA}	1.30 ± 0.13 ^{cA}
	TP	SSF_A_SSHG1	6.85 ± 0.45 ^{aB}	8.04 ± 0.22 ^{aA}	<1.00 ^{bA}	1.18 ± 0.12 ^{cA}	<1.00 ^{cA}	1.60 ± 0.25 ^{cA}
	TP	SSF_A+P_SSHG1	6.85 ± 0.40 ^{aB}	8.12 ± 0.22 ^{aA}	<1.00 ^{bA}	1.18 ± 0.12 ^{cA}	<1.00 ^{cA}	1.25 ± 0.13 ^{cA}
	TP	SSF_A_PDY(S)	6.85 ± 0.33 ^{aB}	8.08 ± 0.30 ^{aA}	<1.00 ^{bA}	1.18 ± 0.12^{cA}	<1.00 ^{cA}	1.30 ± 0.10 ^{cA}
	TP	SSF_A+P_PDY(S)	6.85 ± 0.42 ^{aB}	8.15 ± 0.20 ^{aA}	<1.00 ^{bA}	1.18±0.12^{cA}	<1.00^{cA}	1.25±0.15^{cA}
	TP	SHF_A_SSHG1	6.85 ± 0.40 ^{aB}	8.04 ± 0.25 ^{aA}	<1.00 ^{bA}	1.18 ± 0.12 ^{cA}	<1.00 ^{cA}	1.23±0.18^{cA}
	TP	SHF_A+P_SSHG1	6.85 ±0.63 ^{aB}	8.14 ± 0.25 ^{aA}	<1.00 ^{bA}	1.18 ± 0.12 ^{cA}	<1.00 ^{cA}	1.18 ± 0.13 ^{cA}
	TP	SHF_A_PDY(S)	6.85 ±0.40 ^{aB}	8.06 ± 0.22 ^{aA}	<1.00 ^{bA}	1.18 ± 0.12 ^{cA}	<1.00 ^{cA}	1.22 ± 0.15^{cA}
	TP	SHF_A+P_PDY(S)	6.85 ±0.50 aB	8.04 ± 0.18 aA	<1.00 ^{ba}	1.18 ± 0.12^{cA}	<1.00 ^{cA}	1.15 ± 0.08 ^{cA}
Organic	PLS	SSF_A_SSHG1	6.85 ± 0.39 ^{aA}	6.55 ± 0.40 ^{bA}	1.50 ± 0.20 ^{aB}	4.08 ± 0.63 ^{abA}	1.65 ± 0.15 ^{aB}	4.11 ± 0.63 ^{aA}
	PLS	SSF_A+P_SSHG1	6.85 ± 0.43 ^{aA}	5.45 ± 0.20 ^{bcB}	1.50 ± 0.20 ^{aB}	4.50 ± 0.30 ^{aA}	1.60 ± 0.15 ^{aB}	4.65 ± 0.23 ^{aA}
	PLS	SSF_A_PDY(S)	6.85 ± 0.49 ^{aA}	5.80 ± 0.25 ^{bcB}	1.50 ± 0.20^{aB}	4.30 ± 0.30 ^{abA}	1.62 ± 0.15 ^{aB}	4.45 ± 0.23 ^{aA}
	PLS	SSF_A+P_PDY(S)	6.85 ± 0.40 ^{aA}	5.65 ± 0.20 ^{bcB}	1.50 ± 0.20 ^{aB}	4.60 ± 0.20 ^{aA}	1.58 ± 0.15 ^{aB}	4.65 ± 0.23 ^{aA}
	PLS	SHF_A_SSHG1	6.85 ± 0.44 ^{aA}	5.55 ± 0.40 ^{bcB}	1.50±0.20 ^{aB}	3.80 ± 0.50 ^{bA}	1.60 ± 0.15 ^{aB}	3.95 ± 0.20 ^{bA}
	PLS	SHF_A+P_SSHG1	6.85 ± 0.45 ^{aA}	5.85 ± 0.25 ^{bcB}	1.50 ± 0.20 ^{aB}	4.08 ± 0.63 ^{abA}	1.60 ± 0.15 ^{aB}	4.11 ± 0.63 ^{bA}
	PLS	SHF_A_PDY(S)	6.85 ± 0.40 ^{aA}	5.55 ± 0.40 ^{bcB}	1.50 ± 0.20 ^{aB}	3.50 ± 0.50 ^{bA}	1.65 ± 0.15 ^{aB}	3.65 ± 0.40 ^{bA}
	PLS	SHF_A+P_PDY(S)	6.85 ± 0.32 ^{aA}	5.85 ± 0.25 ^{bcB}	1.50 ± 0.20 ^{aB}	3.70 ± 0.35 ^{bA}	1.60 ± 0.15 ^{aB}	3.80 ± 0.20 ^{bA}
	TP	SSF_A_SSHG1	6.85 ±0.63 ^{aB}	8.04 ± 0.22 ^{aA}	<1.00 ^{bB}	3.75 ± 0.12 ^{bA}	<1.00 ^{cB}	3.80 ± 0.35 ^{bA}
	TP	SSF_A+P_SSHG1	6.85 ±0.42 ^{aB}	8.00 ± 0.20 ^{aA}	<1.00 ^{bA}	1.05 ± 0.22 ^{cA}	<1.00 ^{cA}	1.15 ± 0.10 ^{cA}
	TP	SSF_A_PDY(S)	6.85 ± 0.34 ^{aB}	8.04 ± 0.22 ^{aA}	<1.00 ^{bA}	1.15 ± 0.08 ^{cA}	<1.00 ^{cA}	1.25 ± 0.12 ^{cA}
	TP	SSF_A+P_PDY(S)	6.85 ± 0.42 ^{aB}	8.15 ± 0.15 ^{aA}	<1.00 ^{bA}	1.05 ± 0.20 ^{cA}	<1.00 ^{cA}	1.15 ± 0.10 ^{cA}
	TP	SHF_A_SSHG1	6.85 ± 0.48 ^{aB}	8.05 ± 0.25 ^{aA}	<1.00 ^{bA}	1.00 ± 0.12 ^{cA}	<1.00 ^{cA}	1.15 ± 0.12 ^{cA}
	TP	SHF_A+P_SSHG1	6.85 ± 0.75 ^{aA}	7.95 ± 0.20 ^{aA}	<1.00 ^{bA}	1.10 ± 0.15 ^{cA}	<1.00 ^{cA}	1.18 ± 0.10 ^{cA}
	TP	SHF_A_PDY(S)	6.85 ±0.65 ^{aB}	8.00 ± 0.23 ^{aA}	<1.00 ^{bA}	1.05 ± 0.22 ^{cA}	<1.00 ^{cA}	1.10 ± 0.05 ^{cA}
	TP	SHF_A+P_PDY(S)	6.85 ±0.38 ^{aA}	7.95 ± 0.20 ^{aA}	<1.00 ^{bA}	1.00 ± 0.10 ^{cA}	<1.00 ^{cA}	1.15 ± 0.15 ^{cA}

Different lower-case letters (a-c) in columns designate statistically significant differences ($p < 0.05$) between means (Tukey's test, at a significance level of 0.05).

Different capital letters (A-B) in rows, separately for yeast, LAB and TMB, designate statistically significant differences ($p < 0.05$) between means (Tukey's test, at a significance level of 0.05).

PLS – pressureless starch liberation; TP – thermal-pressure starch liberation; SSF – simultaneous saccharification and fermentation; SHF – separate hydrolysis and fermentation; A – amylolytic enzymes; A+P – amylolytic enzymes + protease; SSHG1 – SafSpirit HG-1 yeast; PDY(S) – Pinnacle Distillers Yeast (S).
